# Origin of the isotropic motion in crystalline molecular rotors with carbazole stators†
†Electronic supplementary information (ESI) available: Synthetic procedures, structural analysis, solid-state ^2^H NMR, solution NMR spectra, PXRD data, computational details. CCDC 1869538–1869541, 1896659. For ESI and crystallographic data in CIF or other electronic format see DOI: 10.1039/c8sc04398a


**DOI:** 10.1039/c8sc04398a

**Published:** 2019-03-20

**Authors:** Abraham Colin-Molina, Marcus J. Jellen, Eduardo García-Quezada, Miguel Eduardo Cifuentes-Quintal, Fernando Murillo, Jorge Barroso, Salvador Pérez-Estrada, Rubén A. Toscano, Gabriel Merino, Braulio Rodríguez-Molina

**Affiliations:** a Instituto de Química , Universidad Nacional Autónoma de México , Circuito Exterior , Ciudad Universitaria , Ciudad de México , 04510 , Mexico . Email: brodriguez@iquimica.unam.mx; b Department of Chemistry and Biochemistry , University of California , Los Angeles , California 90095 , USA; c Departamento de Física Aplicada , Centro de Investigación y de Estudios Avanzados , Unidad Mérida. Km 6 Antigua Carretera a Progreso. Apdo. Postal 73, Cordemex , Mérida , 97310 , Yuc. , Mexico . Email: gmerino@cinvestav.mx; d Área Académica de Química , Centro de Investigaciones Químicas , Universidad Autónoma del Estado de Hidalgo , km 4.5 Carretera Pachuca-Tulancingo, Ciudad del Conocimiento , Mineral de la Reforma , Hidalgo 42184 , Mexico

## Abstract

Herein we report two crystalline molecular rotors **1** and **4** that show extremely narrow signals in deuterium solid-state NMR spectroscopy. Although this line shape is typically associated with fast-moving molecular components, our VT ^2^H NMR experiments, along with X-ray diffraction analyses and periodic DFT computations show that this spectroscopic feature can also be originated from low-frequency intramolecular rotations of the central phenylene with a cone angle of 54.7° that is attained by the cooperative motion of the entire structure that distorts the molecular axis to rotation. In contrast, two isomeric structures (**2** and **3**) do not show a noticeable intramolecular rotation, because their crystallographic arrays showed very restricting close contacts. Our findings clearly indicate that the multiple components and phase transitions in crystalline molecular machines can work in concert to achieve the desired motion.

## Abstract 2

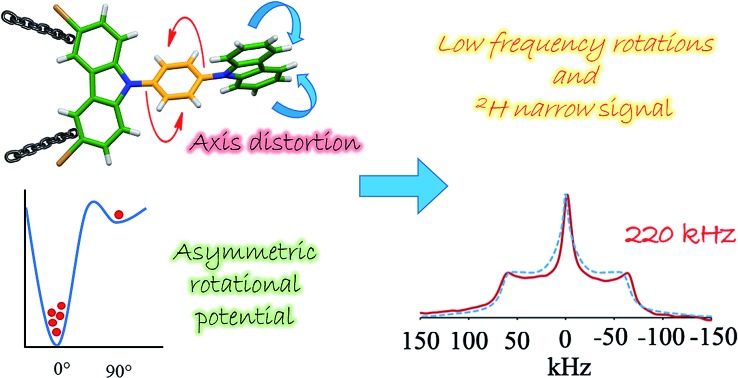
Herein we report two crystalline molecular rotors **1** and **4** that show extremely narrow signals in deuterium solid-state NMR spectroscopy.

## Introduction

Understanding the fundamental mechanisms of motion in artificial molecular machines is a challenging task and the focus of intense research around the globe.^1^ By taking advantage of the dynamics at the molecular level, one can envision numerous technological applications for molecular machines such as responsive sensors or memory devices.^2^ Among other machines at the nanoscale,^3^ crystalline molecular rotors are designed to have angular displacements in the solid-state as the result of the concerted function of two components: (1) a small group is known as a rotator, which shows various degrees of motion within the crystal lattice, and (2) a large and rigid fragment referred to as a stator, intended to direct the crystallization and generate a cavity for the rotation to occur.^4^,^5^

As recently reported by the pioneering groups of Garcia-Garibay,^6^ Batail,^7^ Michl,^8^ and others,^9^ the use of crystal engineering strategies can afford molecular rotors with programmable control of the solid-state dynamics, exhibiting motions that span frequencies ranging from the kilohertz regime up to the ultrafast rotations. Different degrees of rotation can be programmed into a crystal by developing crystalline entities with various architectures and functionalities, for example by means of supramolecular interactions^10^ or by using novel solid-solution approaches.^11^ Drawing from these strategies, we recently reported a crystalline molecular rotor with halogen bonds, which exhibits a very fast and unusual rotation characterized by an extremely sharp deuterium signal,^12^ a spectroscopic feature that is typically found in proton conducting solid materials (phosphonic acids and imidazole-based compounds),^13^ or solvent molecules freely reorienting within crystal lattices.^14^

In crystalline molecular rotors, the frequency, as well as the geometry of the rotation, are the result of the combination of many structural aspects.^15^ In the case of the above-mentioned rotor, the phenylene rotators are sandwiched between carbazole stators bearing peripheral aryl halogen bonds, which allow for either halogen or hydrogen bonded structures to form. Such supramolecular interactions are considered to play a pivotal role in the array of rotors, resulting in a planar conformation which favored the intramolecular dynamics.

**Fig. 1:**
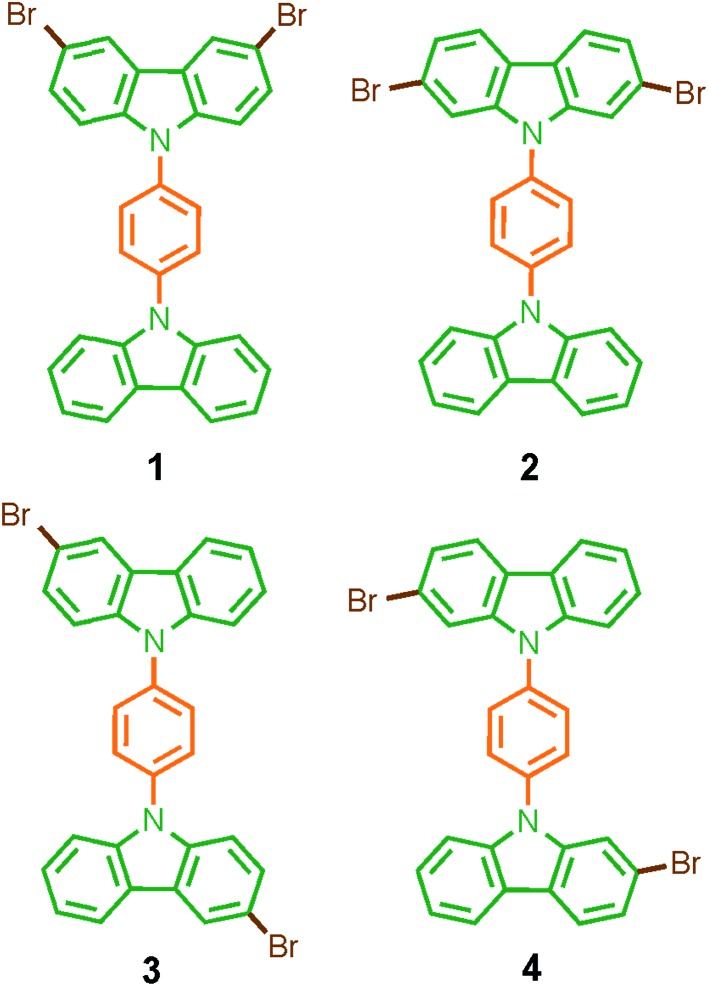
Molecular rotors **1–4** reported here with dibrominated carbazole stators.

Considering that systematic changes in the structure would modify the rotational frequency in similar molecules, we decided to evaluate the influence of the halogen atoms in the molecular conformation and more importantly, in the rotational motion. Therefore, in this work we describe the synthesis, solid-state characterization, and dynamics of four dibrominated bis(carbazole-9-yl)phenylene compounds **1–4** (Fig. 1), each of which features only two bromine atoms in the periphery. The main difference in their design was the position of the halogens in the carbazole stator, which was expected to give rise to very distinctive supramolecular arrays and intramolecular dynamics.

The X-ray analyses of these compounds revealed that three of them adopted a planar conformation, but contrary to the reported tetrabrominated counterpart, the solid-state ^2^H NMR experiments showed a broad line ^2^H shape at room temperature that is characteristic of static molecular components. We then decided to increase the temperature of the samples to induce changes in the spectra, surprisingly the broad line shape of two compounds (**1** and **4**) became extremely narrow above 350 K. At this point, the collected solid-state NMR data was somehow contradicting because the broad Pake pattern suggested the presence of low rotational frequencies, but the previous evidence prompted us to associate the extremely narrow signal with an ultrafast motion. Both of these features appearing on a single spectrum is counter-intuitive and therefore must be arising from an unexpected molecular torsion within the crystal.

The solid-state NMR results encouraged us to perform computational studies, which afforded valuable insights into the mechanism responsible for this unexpected deuterium signal. It is important to note that computations using periodic boundary conditions in crystalline molecular rotors are scarce.^16^–^18^ Regularly, to study such systems a significant simplification is considered, *i.e.* a gas phase computation to determine the rotational barrier without considering the crystal environment, or alternatively, a cluster approach that only involves the first contacts around the molecular rotor. Such considerations could be qualitatively consistent with the experimental data for molecular crystals when there is enough space for rotation. However, in some cases there are significant differences between the experimental and the theoretical values, and therefore it is mandatory to consider a most extensive description of the intermolecular effects in the crystal, that of course becomes computationally expensive. In this work, we performed a series of computations using periodic boundary conditions for a better description of the title molecular rotors compounds, since as it is shown, in some cases the deformation of the crystal units caused by its surroundings, is completely needed to explain the intramolecular rotation.

Our computations indicate that in the cases of rotors **1** and **4**, the carbazoles and phenylene bent to allow the phenyl groups explore 90° jumps, describing an asymmetric rotational potential made of four minima. In the case of the phenylene rings in compounds **1** and **4**, a cone angle of 54.7° is then generated and thus gives rise to the sharp signal. Taking the spectroscopic and computational data together, we propose that these minima and the distortion of the molecular axis at high temperatures are both structural requirements for the narrow deuterium signals to occur, irrespective of the rotational frequency. The findings included here reveal that the synchronized action of the molecular components is at the origin of the rotation of these crystalline rotors.

## Results and discussion

### Synthesis and characterization

**Scheme 1:**
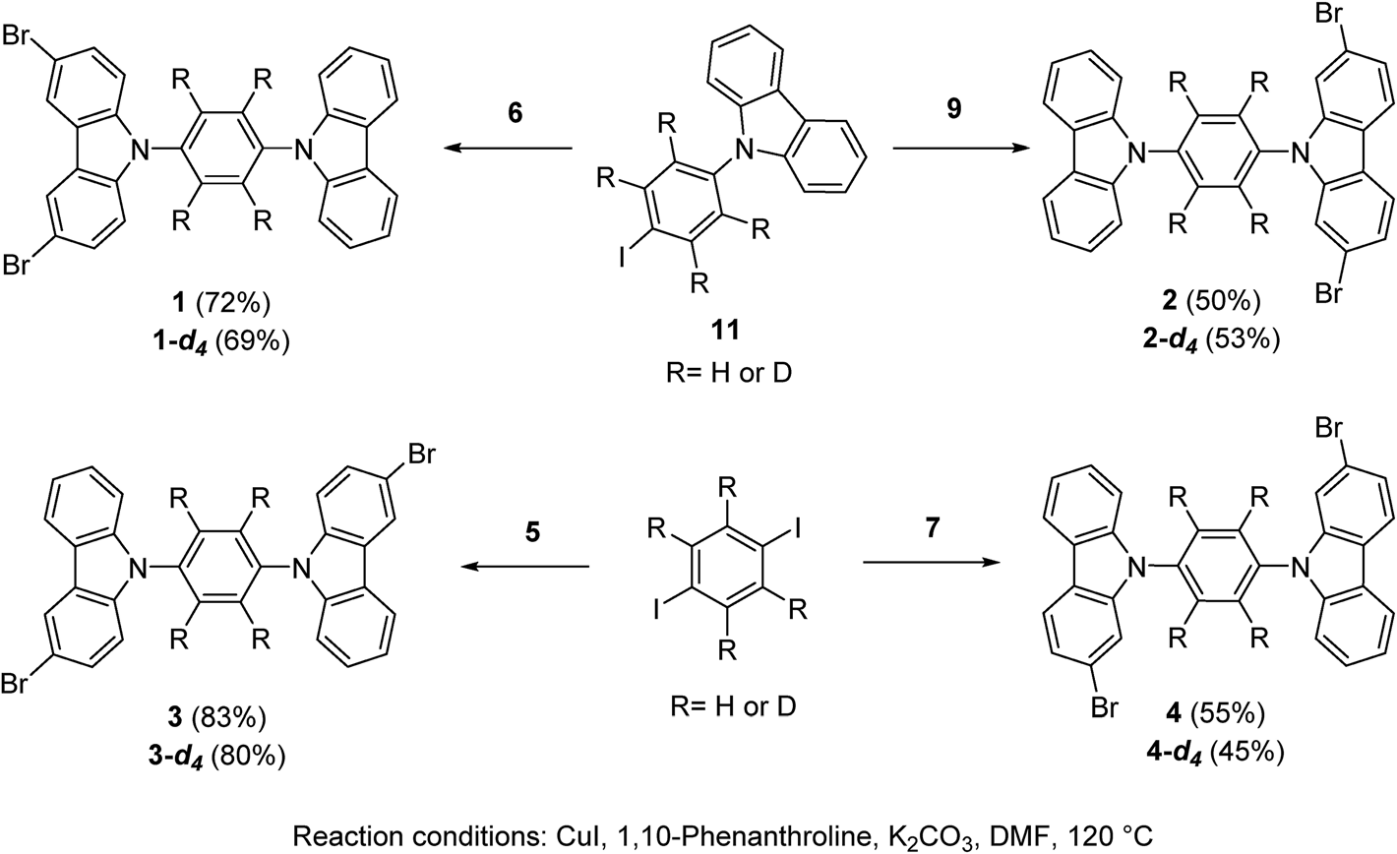
Synthesis of rotors **1–4** and deuterated analogues, see the ESI† for the synthesis of the precursors.

Rotors **1** and **2**, and their deuterated analogs **1-d_4_** and **2-d_4_**, were prepared in moderate to good yields (50% to 72%) by Ullmann coupling between 9-(4-iodophenyl)-carbazole **11** or **11-d_4_** and the corresponding dibrominated carbazole compounds **6** or **9** (Scheme 1). Complementarily, rotors **3** and **4** and their deuterium enriched analogs **3-d_4_** and **4-d_4_** were obtained in similar yields (45% to 83%) by coupling the corresponding carbazol derivatives **5** and **7** and 1,4-diiodobenzene (or 1,4-diiodobenzene-d_4_) (Scheme 1).

The complete spectroscopic characterization (^1^H and ^13^C NMR, MS and FTIR) of all rotors and intermediates can be found in the ESI.†

### Single crystal and powder X-ray diffraction studies of rotors **1–4**

The position of the halogen atoms clearly directed crystallization of all compounds, each showing significantly different molecular conformations in the solid-state, as compared with their parent rotor (ESI Fig. S1†).

**Fig. 2:**
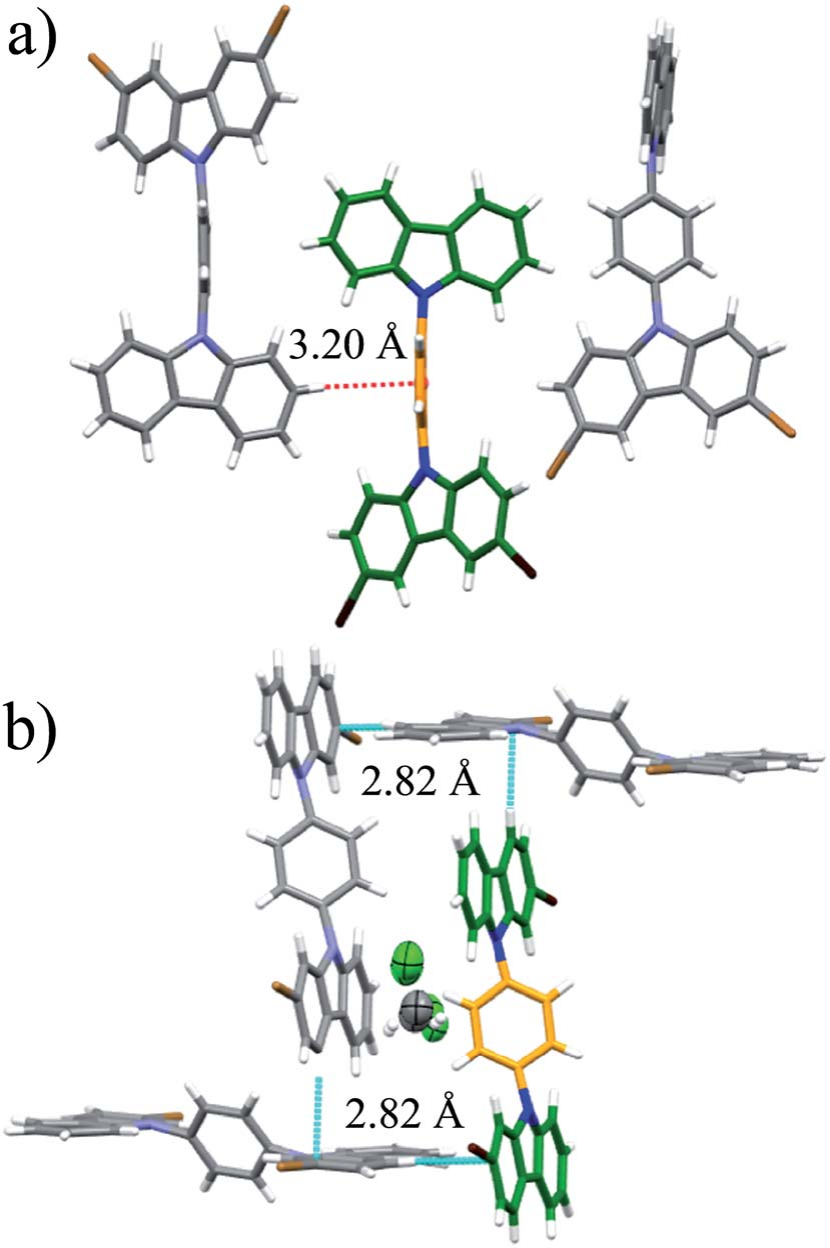
(a) Supramolecular interactions of the molecular rotor **1** and (b) interactions of the solvate of rotor **4**. Both rotors show a sharp deuterium signal at high temperatures.

The rotor **1** adopted a twisted conformation with the surrounding carbazole moieties having dihedral angles of 46.5° and 58.0° with respect to the central phenylene. The crystalline array is governed by a non-conventional C–H···Br hydrogen bond, with an H···Br distance of 3.01 Å and a donor–H–acceptor angle (C–H···Br) of 145.8°. This hydrogen bond is noticeably shorter and more directional than the one found in 1,4-dibromobenzene (H···Br distance: 3.13 Å, donor–H–acceptor angle (C–H···Br): 148.91°).^19^ In addition, the brominated-carbazole fragments displayed an antiparallel π-stacking along the crystallographic *a*-axis, with an interplanar distance of 3.55 Å (ESI, Fig. S2†). The inner phenylene of molecular rotor **1** experiences C–H···π interactions from adjacent carbazole stators (Fig. 2a). These contacts are usually considered as weak,^20^ and thus, should not interfere with intramolecular motion of this rotor.

Compound **2** adopted a planar conformation with the central phenylene showing a dihedral angle of *ca.* 53° with respect to the surrounding carbazole fragments. The crystal array of **2** involves two types of intermolecular contacts, non-conventional C–H···Br hydrogen bonds and π-stacking interactions within the non-brominated fragments. The central phenylene is flanked by two bromine atoms, which impose substantial steric hindrance and therefore no internal motion can be expected (Fig. S3†). The quasi-planar molecules in the crystalline array of **3** do not reveal π-stacking. The halogen atoms participate in a hydrogen bond C–H···Br with a distance of 3.02 Å and a donor–H–acceptor angle of 124.8°. The central phenylene has two close contacts with neighboring carbazole stators, and therefore the rotation may be restricted (Fig. S4†).

For rotor **4**, we grew a solvate from dichloromethane (Fig. 2b). Interestingly, the molecule also adopts a planar conformation even with the guest molecules in the crystal. The main intermolecular interactions found in this crystal are C–H···π interactions between neighboring carbazole stators, with a distance of 2.82 Å (Fig. 2b and S5†), resulting in a perpendicular array of rotors. It seemed reasonable that the entrapped dichloromethane molecules should impose a significant steric effect over the rotational trajectory of the central phenylene. Interestingly, when crystals grown from dichloromethane are kept at room temperature in an open vial, they become opaque in three days, but they still diffract showing the same packing but without the entrapped solvent (see ESI Fig. S6†).

**Fig. 3:**
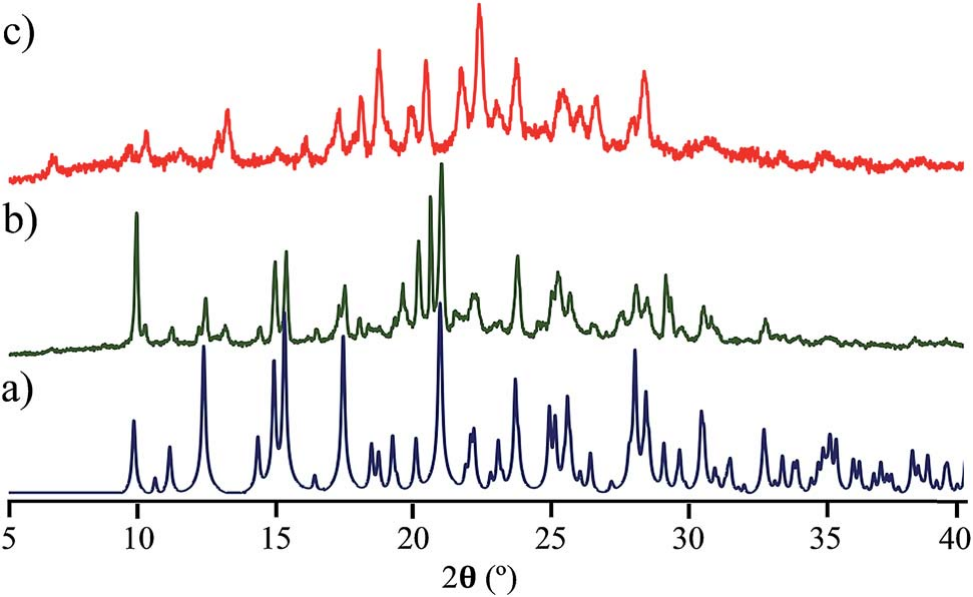
Comparison of the powder X-ray diffraction diffractograms of compound **4**, (a) calculated data starting from the solvent-free structure, (b) experimental data obtained from crystals kept at room temperature over three days, (c) experimental data obtained by heating the sample above 165 °C, see details in the main text.

Interestingly, the loss of solvent does not cause important changes in the crystal parameters as indicated in Table S1.† This desolvation at room temperature was also corroborated by powder X-ray diffraction experiments as indicated in Fig. 3a and b. A Le Bail fitting of the data shows that the diffractogram obtained from aged crystals share more similarities to that calculated from solvent-free structure, with some residual peaks from the previous solvate form (see ESI Fig. S44†). When this desolvated sample is heated above 165 °C, it transforms into a third solid which was unable to be diffracted and thus no single crystal has been obtained. This final solid transformation was further corroborated placing freshly grown crystals under the heating surface of the microscope (see ESI Fig. S45†).

### Solid-state ^2^H NMR and thermal stabilities

Solid-state quadrupolar echo ^2^H NMR spectroscopy provides valuable insight into both the mode and internal dynamics at the molecular level.^21^ This method relies on detecting the orientation-dependence of a C–D bond vector rotating in the presence of an external magnetic field. A doublet will be visible in an NMR spectrum corresponding to every angle of the C–D bond with respect to the magnetic field. For a powdered sample occupying every orientation, a broad signal known as Pake pattern will result. Such patterns experience changes when the groups that contain the C–D bond undergo site-exchange events and the frequency of this motion can be obtained by fitting the experimental spectra with freely-accessible software.^22^

With this in mind, we synthesized the corresponding deuterated rotors **1-d_4_** to **4-d_4_** for solid-state ^2^H NMR experiments (*vide supra*). Bulk solid samples were freshly prepared following the crystallization procedures detailed above, yielding the same crystallographic phase of their natural abundance counterparts, as verified by powder X-ray diffraction experiments (ESI Fig. S38–S42†). These samples were also studied by thermogravimetric analyses (TGA) and differential scanning calorimetry (DSC). Gratifyingly, rotors **1–3** show a very high thermal stability, with a melting point of 230 °C for compound **1**, 311 °C for compound **2** and 292 °C for compound **3** (ESI Fig. S34–S36†). Expectedly, freshly grown samples of the dichloromethane solvate **4** had much lower stability, showing a noticeable desolvation that starts below 100 °C accompanied by the mentioned phase transition, followed by a sharp melting point at 297 °C (ESI, Fig. S37†).

Once the stabilities and the phase identity of the deuterated analogs were determined, we carried out solid-state quadrupolar echo ^2^H NMR^23^ at 295 K. All compounds at this temperature showed the characteristic broad Pake pattern,^24^ which is indicative of immobile phenylenes. Intuitively, we then increased the temperature of the samples to observe changes in the spectra resulting from new molecular dynamics. Despite this temperature increment, compounds **2** and **3** did not present significant changes in the spectra, and therefore, we concluded that achieving motions in these samples was beyond the temperature limits of our instrumentation (see ESI Fig. S7†).

**Fig. 4:**
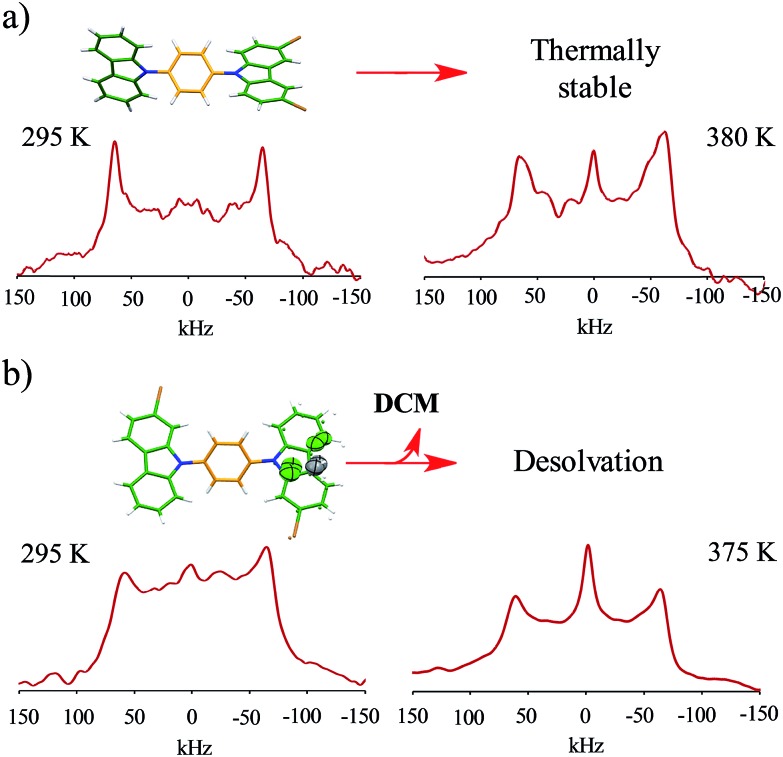
Experimental ^2^H NMR line shapes of (a) compound **1** and (b) compound **4** at the indicated temperatures.

Interestingly, heating compounds **1** and **4** above 350 K gave rise to a very narrow peak in the center of the static deuterium spectra (Fig. 4), an interesting line shape that is usually attributed to isotropic motion in other molecules. These results were indeed surprising because this signal could be taken as indicative of a fast exchanging process, *i.e.*, evidencing very high rotational frequencies, as was the case in our previously reported rotor. Nevertheless, fast rotation in the current rotors **1** and **4** was not considered very likely, as the Pake pattern does not change in an expected fashion for a phenylene rotor, and therefore another mechanism should be involved in the generation of the narrow ^2^H NMR signal.

### Computational studies

To gain insights of the structural requirements that originate the deuterium sharp signal in these rotors, we performed a series of periodic DFT computations on the available X-ray structures (see the Computational details in the ESI†).^25^ Given the complexity of the crystal arrays, the rotation of the phenylene groups was modeled by using two approaches: (1) fixing the positions of the carbazole stators, and (2) considering a full relaxation of the carbazole moieties, which can be seen as the degree of flexibility of the frameworks. Notably, the selected methodology (PBE-D3 26 using a PAW^27^ approach, see the computational details) was able to reproduce the observed structural parameters of compounds **1–4** with errors less than 4.5% (see Table S2†). Two possible angular trajectories within the crystal were examined, *i.e.*, all the phenylenes simultaneously rotate in the same direction (conrotatory) or the opposite direction (disrotatory).

**Fig. 5:**
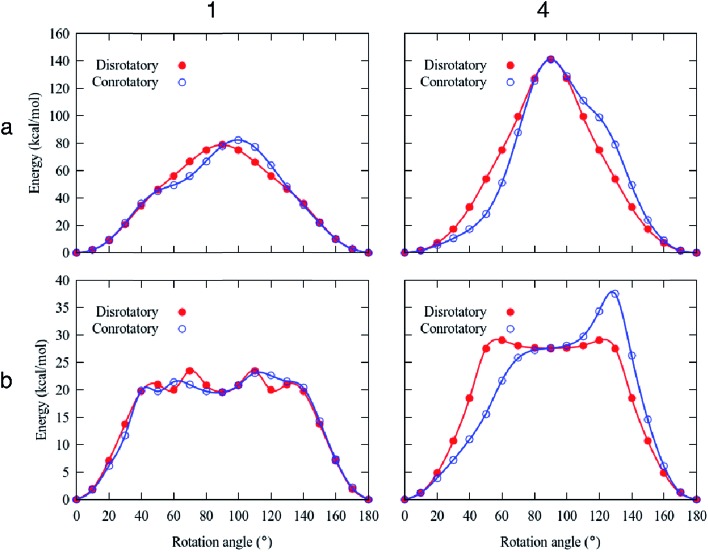
Rotational barriers for compounds **1** and **4**. (a) Rigid model; (b) relaxed model.

High rotational barriers for compounds **1** and **4** (*E*_a_ = 78.8 and 141.0 kcal mol^–1^, respectively) were obtained by fixing the carbazole stators in the conrotatory process (Fig. 5). Similar barriers were computed for the disrotatory motions. The most noticeable difference between the rotational pathways was the presence of a shoulder in the rotational potential diagram. Interestingly, when the relaxation of the carbazole stator was allowed, the barriers decreased drastically. The differences of the computed barriers between the fixed and relaxed models are that in the latter, the phenylene rotator experiences less steric impediment, given that the carbazole components swing away from the central ring allowing additional angular motion. In this way, the molecular components (the central ring and the surrounding carbazole portions) deviate from the molecular axis, resulting in a lower energy transition barrier than that obtained from the rigid model.

For the molecular rotor **1**, both rotational paths conrotatory or disrotatory became viable, showing barriers of 23.5 kcal mol^–1^, which can be surpassed at high temperatures to enable motion. Our computational studies also indicated the presence of the global minima at 0° and 180° and at least one local minimum at *ca.* 90° within the rotational potential (Fig. 5b).

In the case of molecular rotor **4**,^28^ our computations showed that the disrotatory dynamic process is the most probable one. The rotational potential also shows the global minima at 0° and 180° and a local minimum at *ca.* 90°, with a higher barrier *E*_a_ = 29.1 kcal mol^–1^. Our findings indicate that the structural distortion mitigates the repulsions between adjacent molecular components, allowing the molecular axis to bend during the rotation of the phenylene (ESI Videos SV1 and SV2†).

### Dynamic model and the origin of the sharp ^2^H NMR signal in rotors **1** and **4**

Based on the evidence presented above, we next focused on a model that can explain the origin of the narrow signal. The dynamic model that we propose has three key characteristics: (i) it considers that the phenylene explores angular displacements of 90° (four-fold rotation) as supported by the DFT studies, (ii) it takes into account an asymmetric rotational potential with an asymmetric distribution of population (45 : 5 : 45 : 5), and (iii) it undergoes a distortion of the rotational axis, based on the bending of the whole molecule proposed by the computational studies. These are represented as variations of the cone angle, from 60° to of 54.7°.^29^ These three characteristics were crucial to simulate the deuterium spectra of rotors **1** and **4** with excellent agreement (Fig. 4 and S8†), indicating that these rotators experience low rotational frequencies at the explored temperatures. Alternative models using other rotational potentials (Fig. S9†) were explored but showed unsuccessful agreement.

**Fig. 6:**
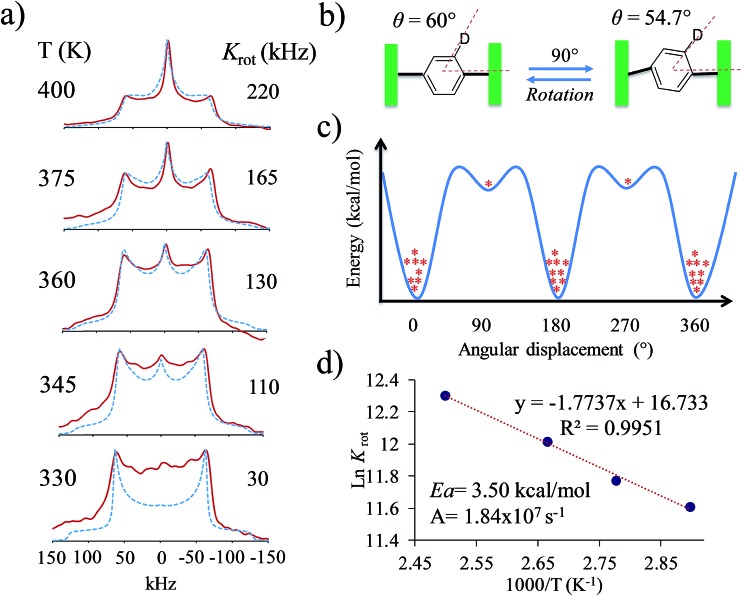
(a) Comparison of the experimental and calculated ^2^H line shapes, (b) distortion of the molecular axis, (c) representation of the asymmetric rotational potential and (d) Arrhenius plot with resulting activation parameters.

By using the proposed dynamic model, we were able to simulate line shapes that reproduce the gradual changes observed for rotor **4** (Fig. 6). An Arrhenius plot using ln *K*_rot_*vs.* 1000/*T* allowed us to determine the activation parameters of the dynamic process. The activation energy to rotation (*E*_a_ = 3.5 kcal mol^–1^) and the pre-exponential factor (*A* = 1.84 × 10^7^ s^–1^) showed low values. Compared to the calculated energy (*E*_a_ = 29.1 kcal mol^–1^), the *E*_a_ obtained from the Arrhenius plot can be explained in terms of the limited thermal stability of the solvate, which above 360 K experiences the exit of the dichloromethane molecules to produce another crystalline solid with increased flexibility of the molecular components. However, although the *E*_a_ is low, it is not possible to achieve fast rotation because the process requires a highly correlated transition state, as indicated by the very low pre-exponential factor.

In the case of the other two rotors, the steric hindrance of the halogen atoms that surround the central phenylene of **2** can justify the high rotational barrier *E*_a_ = 141.6 kcal mol^–1^ (obtained using the relaxed model), explaining why the phenylene rotation is not observed by solid-state NMR spectroscopy. Finally, the results of the compound **3** are contradictory, because the computed rotational barrier for the disrotatory process is *E*_a_ = 23.94 kcal mol^–1^ and thus it was expected to show some indications of intramolecular motion. Unfortunately, the rotation was not observed by solid-state ^2^H NMR. One possible explanation could be that the crystal structure could show additional changes in the unit cell parameters without undergoing a phase transition and that can strengthen some intermolecular interactions, disabling the potential motion of the central phenylene. Those possible structural changes were not considered in our computations.

## Conclusions

In summary, we have synthesized four new dibrominated compounds **1–4** where the position of the bromine atoms had a deep impact in the supramolecular interactions within their crystals, mostly displaying weak C–H···Br interactions. Interestingly, crystalline rotors **1** and **4** showed a distinctive narrow deuterium line shape above 370 K. These results were supported by periodic DFT computations, which showed that the carbazole stators experience an important structural distortion that favored the rotation over an asymmetric rotational potential. Our findings revealed that the extremely narrow ^2^H signals from rotors **1** and **4** originate from low-frequency motions (between 30 and 220 kHz) along a distorted molecular axis to rotation (cone angle of 54.7°), exploring multisite angular displacements (90° jumps), averaging the anisotropic components of the line shape showing only the isotropic contribution.

Particularly in the case of the rotor **4**, a solvate-solvent free phase transition generates a crystalline solid with an environment that facilitates the extremely narrow deuterium signal, but unfortunately, the resulting specimen was small and not susceptible to single crystal X-ray diffraction. Based on the solid state NMR and DFT results, we were able to propose a dynamic model that takes into account the concerted motion, where the rotators explore low energy asymmetric pathways, enabling rotation in environments with high steric congestion. We consider that our findings contribute to deepening knowledge of the function of crystalline molecular machines in which multiple lattice components can work in concert to induce motion.

## Conflicts of interest

The authors declare no competing interests.

## Supplementary Material

Supplementary information

Supplementary movie

Supplementary movie

Crystal structure data
