# Supplementary material for: Origin of the isotropic motion in crystalline molecular rotors with carbazole stators
Source: Chem Sci. 2019 Mar 20;10(16):4422–9. doi: 10.1039/c8sc04398a (PMC6482440; doi:10.1039/c8sc04398a)
Supplement: Supplementary file 1 [file SC-010-C8SC04398A-s001.pdf]

Electronic Supplementary Material (ESI) for Chemical Science.
This journal is © The Royal Society of Chemistry 2019

Supplementary Information

Origin of the Isotropic Motion in Crystalline Molecular Rotors with Carbazole Stators

Abraham Colin-Molina,[1] Marcus Jellen,[2] Eduardo García-Quezada,[1] Eduardo Cifuentes-Quintal,[3] Fernando Murillo,[3] Jorge Barroso,[3] Salvador Pérez-Estrada,[4] Rubén A. Toscano,[1] Gabriel Merino[3] and Braulio Rodríguez-Molina[1]**

[1]Instituto de Química, Universidad Nacional Autónoma de México, Circuito Exterior, Ciudad Universitaria, Ciudad de México, 04510, México.

[2]Department of Chemistry and Biochemistry, University of California, Los Angeles, California 90095, United States.

[3]Departamento de Física Aplicada, Centro de Investigación y de Estudios Avanzados, Unidad Mérida. Km 6 Antigua Carretera a Progreso. Apdo. Postal 73, Cordemex, 97310, Mérida, Yuc., México.

[4]Área Académica de Química, Centro de Investigaciones Químicas, Universidad Autónoma del Estado de Hidalgo, km 4.5 Carretera Pachuca-Tulancingo, Ciudad del Conocimiento, Mineral de la Reforma, Hidalgo 42184, México.

E-mail: gmerino@cinvestav.mx, brodriguez@iquimica.unam.mx

Contents

Materials and methods 2

Experimental section 3

X-Ray diffraction studies 9

^{2}H NMR quadrupolar echo spin in solid state 15

Nuclear Magnetic Resonance of ^{1}H and ^{13}C in solution 17

Thermal analyses 41

Powder X-Ray diffraction 43

Computational Details 46

Materials and methods

All reagents were purchased from Sigma-Aldrich and used as received. The THF was dried prior to use by distillation over Na°/benzophenone. Flash column chromatography was performed using silica gel Aldrich 230-400 mesh. Reactions were monitored by TLC on silica gel plates 60 F_{254} (Merck) and spots were detected either by UV-absorption or by using the Seebach's TLC stain. ^{1}H and ^{13}C NMR data for all compounds were recorded at ambient temperature using Bruker Fourier300, Jeol Eclipse 300 and Bruker AV700 with cryoprobe spectrometers and are referenced to $CDCl_3$ (7.26 ppm, 77.0 ppm), as indicated. The FT-IR spectral data were recorded with Bruker ATR in the 450-4000 cm^{-1} range. Melting points were determined using Fisher Johns melting point apparatus (uncorrected) or by Differential Scanning Calorimetry. HRMS were obtained by Direct Analysis in Real Time (DART) in AccuTOF, JMS-T100LC.

Experimental section

General procedure for the synthesis of rotors 1, 1-*d*$_4$, 2 and 2-*d*$_4$

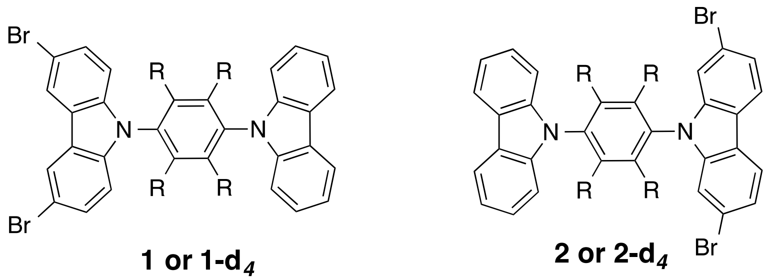

Compounds 1 and 2. 0.290 g of 3,6-dibromo-*9H*-carbazole or 2,7-dibromo-*9H*-carbazole (1.1 eq, 0.89 mmol), 0.300 g of 1-(carbazole-9-yl)-4-iodobenzene (1.0 eq, 0.81 mmol), 0.015 g of CuI (0.1 eq, 0.08 mmol), 0.014 g of 1,10-phenantroline (0.1 eq, 0.08 mmol) and 0.123 g of K_2CO_3 (1.1 eq, 0.89 mmol) were dissolved in 3 mL of anhydrous *N*,*N*-dimethylformamide and heated to 120 ºC under atmosphere of N_2 for 12 h. The reaction crude was poured into an ammonium chloride solution and the solid was filtered and washed with distillated water.

Compound 1. It was purified by column chromatography using silica gel and hexanes as eluent. The product was isolated as a white solid (0.331, yield 72%, m.p. 230 ºC by DSC). ^{1}H NMR (300 MHz, $CDCl_3$) δ: 8.43 (s, 1H), 8.23 (s, 1H), 8.18 (d, *J*=8.0 Hz, 2H), 7.79 (dd, *J*=8.0, 8.0 Hz, 5H), 7.65-7.28 (m, 9H). ^{13}C NMR (75 MHz, $CDCl_3$) δ: 140.6, 137.4, 135.5, 135.1, 129.6, 128.5, 128.3, 126.2, 123.7, 123.4, 120.5, 120.4, 113.5, 112.0, 111.4, 109.7 FT-IR (ATR, cm^{-1}) ν= 3082, 3045, 1514, 1469, 1433, 1315,1228, 797, 746, 723. HRMS (DART) $C_{30}H_{19}Br_2N_2$ m/z calc= 566.98945, found=566.98917 diff. (ppm)= 0.49.

Compound 2. The solid was suspended in boiling ethanol and filtered to obtain the product as a white solid (0.230 g, yield 50 %, m.p. 311.0 ºC by DSC). RMN ^{1}H NMR (700 MHz,

$CDCl_3$) δ: 8.81 (d, J=8.0 Hz, 2H), 8.01 (d, J=8.0 Hz, 2H), 7.89 (d, J=8.0 Hz, 2H), 7.78 (d, J=8.0 Hz, 2H), 7.67 (s, 2H), 7.62 (d, J=8.0 Hz, 2H), 7.53 (t, 2H), 7.48 (d, J=8.0 Hz, 2H), 7.38 (t, 2H). ^{13}C NMR (175 MHz, $CDCl_3$) δ: 141.8, 140.6, 137.71, 136.84, 135.2, 128.7, 128.5, 126.2, 124.0, 123.7, 121.9, 121.6, 120.4, 120.1, 113.0, 109.8. FT-IR (ATR, cm^{-1}) ν= 3129, 3042, 1674, 1514, 1226, 588, 512. HRMS (DART) $C_{30}H_{19}Br_2N_2$ m/z calc= 566.98945, found=566.98952 diff. (ppm)=0.11

Procedure for compounds 1-*d₄* and 2-*d₄*. For the preparation of these compounds was followed the procedure indicated for their analogues no deuterated. The quantities employed are the next: 0.287 g of 3,6-dibromo-*9H*-carbazole or 2,7-dibromo-*9H*-carbazole (1.1 eq, 0.88 mmol), 0.300 g of 1-(carbazole-9-yl)-4-iodobenzene-d_4 (1.0 eq, 0.80 mmol), 0.015 g of CuI (0.1 eq, 0.08 mmol), 0.014 g of 1,10-phenantroline (0.1 eq, 0.08 mmol) and 0.122 g of K_2CO_3 (1.1 eq, 0.88 mmol).

Compound 1-d_4. White powder (0.312 g, yield 69%). ^1H NMR (300 MHz, $CDCl_3$) δ: 8.23 (d, J=2.0 Hz, 2H), 8.19 (d, J= 8.0 Hz, 2H), 7.55-7.30 (m, 10H). ^{13}C NMR (75 MHz, $CDCl_3$) δ: 140.7, 137.3, 135.6, 135.2, 129.7, 126.3, 123.7, 123.5, 120.6, 120.5, 113.5, 112.1, 111.6, 109.8. IR-FT (ATR, cm^{-1}) ν= 3052, 1470, 1309, 1282, 1228, 798, 746, 722. HRMS (DART) $C_{30}H_{15}D_4Br_2N_2$ m/z calc= 571.01456, found=571.01472, diff. (ppm)=0.29.

Compound 2-d_4. White powder (0.242 g, yield 53 %). ^1H NMR (300 MHz, $CDCl_3$) δ: 8.21 (d, J= 8.0 Hz, 2H), 8.01 (d, J= 8.0 Hz, 2H), 7.66-7.61 (m, 2H), 7.55-7.40 (m, 2H), 7.35 (t, 1H). ^{13}C NMR (75 MHz, $CDCl_3$) δ: 141.8, 140.6, 137.6, 136.8, 135.1, 126.2, 124.0, 123.7, 121.9, 121.6, 120.4, 120.2, 113.0, 109.7. IR-FT (ATR, cm^{-1}) ν= 3045, 1584, 1482, 1451,

1327, 1225, 941, 738, 717. HRMS (DART) $C_{30}H_{15}D_4Br_2N_2$ m/z calc= 571.01456, found=571.01188, diff. (ppm)= 4.7

General procedure for the synthesis of rotors 3, 3-*d₄*, 4 and 4-*d₄*

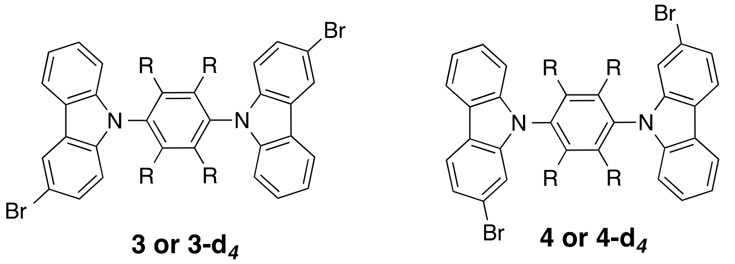

3 or 3-d$_4$ **4 or 4-d$_4$**

Compounds 3 and 4. 0.492 g of 3-bromo-*9H*-carbazole or 2-bromo-*9H*-carbazole (2.2 eq, 2.00 mmol), 0.300 g of 1,4-diiodobenzene (1.0 eq, 0.91 mmol), 0.034 g of CuI (0.2 eq, 0.18 mmol), 0.032 g of 1,10-phenantroline (0.2 eq, 0.18 mmol) and 0.276 g of K_2CO_3 (2.2 eq, 2.00 mmol) were dissolved in 4 mL of anhydrous *N*,*N*-dimethylformamide and heated to 120 ºC under atmosphere of N_2 for 8 h. The crude in every case was poured into a saturated ammonium chloride solution and the solid was filtered.

Compound 3. The product was suspended in boiling ethanol and the solid obtained was filtered giving a white solid as product (0.427 g, yield 83%, m.p. 292.6 ºC by DSC). ^{1}H NMR (300 MHz, $CDCl_3$) δ: 8.29 (s, 2H), 8.13 (d, *J*=8.0 Hz, 2H), 7.80 (s, 4H), 7.60-7.30 (m, 10H). ^{13}C NMR (75 MHz, $CDCl_3$) δ: 141.1, 139.5, 136.6, 128.9, 128.5, 127.0, 125.5, 123.3, 122.6, 120.9, 113.2, 111.3, 110.0. IR-FT (ATR, cm^{-1}) ν= 3045, 1514, 1466, 1441, 1267, 1229, 795, 767, 747. HRMS (DART) $C_{30}H_{19}Br_2N_2$ m/z calc= 566.99150, found=566.99085, diff. (ppm)= 1.14.

Compound 4. The product was purified by column chromatography using hexanes/dichloromethane (97:3) as elution system. Title compound was isolated as a white solid (0.283 g, yield 55%, m.p. 297.4 ºC by DSC). ^{1}H NMR (300 MHz, $CDCl_3$) δ: 8.17 (d,

J=8.0 Hz, 2H), 8.05 (d, *J*=8.0 Hz, 2H), 7.83 (s, 4H), 7.71 (d, *J*=2.0 Hz, 2H), 7.54 (d, *J*=8.0 Hz, 4H), 7.47 (dd, *J*=8.0, 2.0 Hz, 2H), 7.38 (t, 2H). ^{13}C NMR (75 MHz, $CDCl_3$) δ: 141.5, 140.1, 136.5, 128.6, 126.6, 123.6, 123.0, 122.6, 121.6, 120.8, 120.4, 119.8, 112.8, 110.0. IR-FT (ATR, cm^{-1}) ν= 3129, 3042, 1674, 1514, 1226 588, 512. HRMS (DART) $C_{30}H_{19}Br_2N_2$ m/z calc= 564.99150, found=564.99011, diff. (ppm)= 2.46

Compounds 3-d₄ and 4-d₄. These compounds were synthesized and poured like is described for their analogues no deuterated. 0.482 g of 3-bromo-*9H*-carbazole or 2-bromo-*9H*-carbazole (2.2 eq, 1.97 mmol), 0.300 g of 1,4-diiodobenzene-d_4 (1.0 eq, 0.89 mmol), 0.034 g of CuI (0.2 eq, 0.17 mmol), 0.032 g of 1,10-phenantroline (0.2 eq, 0.17 mmol) and 0.272 g of K_2CO_3 (2.2 eq, 1.97 mmol).

Compound 3-*d*$_4$. White powder (0.409 g, yield 80%). ^{1}H NMR (300 MHz, $CDCl_3$) δ: 8.28 (s, 2H), 8.12 (d, *J*=8.0 Hz, 2H), 7.60-7.32 (m, 10H). ^{13}C NMR (75 MHz, $CDCl_3$) δ: 140.6, 139.0, 128.4, 126.5, 125.0, 122.9, 122.1, 120.4, 120.3, 112.7, 110.8, 109.5. IR-FT (ATR, cm^{-1}) ν= 3055, 1489, 1454, 1267, 1228, 797. HRMS (DART) $C_{30}H_{15}D_4Br_2N_2$ m/z calc=571.01456, found=571.01367, diff. (ppm)=1.55.

Compound 4-*d*$_4$. White powder (0.231 g, yield 45 %). ^{1}H NMR (300 MHz, $CDCl_3$) δ: 8.17 (d, *J*=8.0 Hz, 2H), 8.05 (d, *J*=8.0 Hz, 2H), 7.71 (d, *J*=2.0 Hz, 2H), 7.54 (d, *J*=8.0 Hz, 4H), 7.47 (dd, *J*=8.0, 2.0 Hz, 2H), 7.38 (t, 2H). ^{13}C NMR (75 MHz, $CDCl_3$) δ: 141.5, 140.9, 136.3, 126.6, 123.5, 123.0, 122.6, 121.6, 120.8, 120.4, 119.8, 112.8, 110.0. IR-FT (ATR, cm^{-1}) ν= 3050, 1586, 1467, 1447, 1327, 1226, 746, 720. HRMS (DART) $C_{30}H_{15}D_4Br_2N_2$ m/z calc=571.01456, found=571.01695, diff. (ppm)=4.18

Synthesis of intermediates

Scheme S1. *Synthetic procedure to obtain bromo-substituted carbazoles*

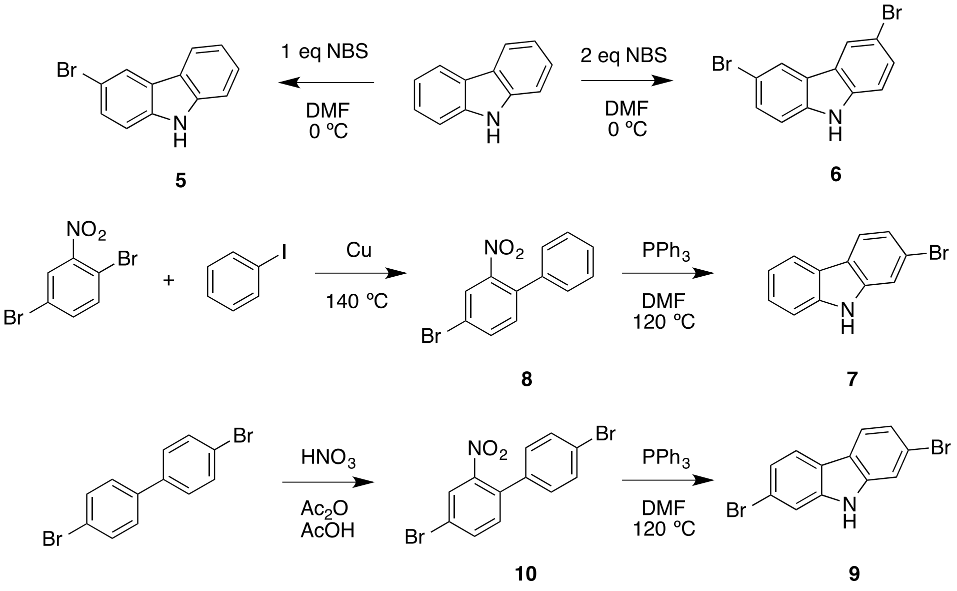

General procedure for the synthesis of 3-Bromo-9*H*-carbazole (5) and 3,6-Dibromo-9*H*-carbazole (6). Compounds **5** and **6** were prepared following reported procedures.[1,2] 0.53 g (2.99 mmol) or 1.06 g (5.98 mmol) of NBS dissolved in DMF were added to a solution of carbazole (0.5 g, 2.99 mmol) in DMF to give **5** (yield 80 %) and **6** (yield 97 %) respectively. Spectroscopic data matched with the previously reported.

4-Bromo-2-Nitro-1,1'-biphenyl (8). 2,5-dibromo-1-nitrobenzene (0.100 g, 0.36 mmol) and copper powder (0.079 g, 1.25 mmol) were added to iodobenzene (3 mL) and heated at 140 ºC overnight. The reaction mixture was cooled to room temperature and filtered under silice using 10 mL of ethyl acetate. The crude was purified by silice column chromatography using hexanes as eluent and the product was isolated as a yellow oil (0.081 g, yield 82%) ^{1}H NMR (300 MHz, $CDCl_3$) δ: 8.02 (d, *J*=2.0 Hz, 1H), 7.76 (dd, *J*=8.3, 2.0 Hz, 1H), 7.46-7.44 (m, 3H), 7.36-7.30 (m, 3H). ^{13}C NMR (75 MHz, $CDCl_3$) δ: 149.6, 136.3, 135.4, 135.2, 133.3,

128.9, 128.6, 127.8, 127.0, 121.3. FT-IR (ATR, cm^{-1}) ν= 3089, 2862, 1549, 1518, 1345, 810. HRMS (DART) $C_{12}H_9BrNO_2$ m/z calc.= 277.98167, found= 277.98147, diff. (ppm)= 0.70

4,4'-dibromo-2-nitro-biphenyl (10). 4,4'-dibromobiphenyle (0.500 g, 1.8 mmol) was suspended in 20 mL of a mixture of acetic acid/acetic anhydride (1:5) and 6 mL of nitric acid was added slowly at 0 ºC. The reaction mixture was stirred for 3 h from 0 ºC to room temperature and the solid was filtered and recrystallized from ethanol to give a pale-yellow powder (0.560 g, yield 98%, m.p. 119-122 ºC). ^{1}H NMR (300 MHz, $CDCl_3$) δ: 8.02 (d, *J*=2.0 Hz, 1H), 7.74 (dd, *J*=8.0, 2.0 Hz, 1H), 7.55 (d, *J*=8.0, 2H), 7.28 (d, *J*=8.0, 1H), 7.14 (d, *J*=8.0, 2H) ^{13}C NMR (75 MHz, $CDCl_3$) δ: 149.2, 135.5, 135.3, 134.1, 133.0, 132.0, 129.4, 127.2, 123.0, 121.8. FT-IR (ATR, cm^{-1}) ν= 2000, 1600, 1537, 1334, 1070. HRMS (DART) $C_{12}H_8Br_2NO_2$ m/z calc= 357.89013, found=357.88961, diff. (ppm)=1.46

General procedure for the synthesis of 2 and 7 bromo-substituted carbazole (7 and 9) from nitro-biphenyl. 0.5 g of nitro-biphenyle derivative (**8**) or (**10**) and 2 eq of triphenylphosphine were added to *N*,*N*-dimethylformamide (3 mL) and heated at reflux for 5 hours under atmosphere of N_2. The reaction mixture was poured into saturated ammonium chloride solution and extracted with dichloromethane (3x10 mL), products were purified by silice column chromatography using hexanes/dichloromethane as eluent and the carbazole bromo-substituted were isolated as white powders.

2-Bromo-9*H*-carbazole (7) (0.376 g, yield 85%, m.p. 124-127 ºC) ^{1}H NMR (300 MHz, $CDCl_3$) δ: 8.01-7.91 (m, 2H), 7.85 (d, *J*=8.0Hz, 1H), 7.51 (d, *J*=2.0 Hz, 1H), 7.40-7.32 (m, 2H), 7.27 (dd, *J*=8.0, 2.0 Hz, 1H), 7.22-7.13 (m, 2H). ^{13}C NMR (75 MHz, $CDCl_3$) δ: 140.3, 139.6, 126.3, 122.8, 122.7, 122.4, 121.5, 120.3, 120.0, 119.3, 113.6, 110.7. FT-IR (ATR,

cm^{-1}) ν= 3386, 3052, 1594, 1433, 1320, 1050. HRMS (DART) $C_{12}H_9BrN$ m/z calc= 245.99246, found= 245.99184, diff. (ppm)=2.55

2,7-dibromo-9*H*-carbazole (9) (0.327 g, yield 72%, m.p. 151-154 ºC) ^{1}H NMR (300 MHz, $CDCl_3$) δ: 8.03 (s, 1H), 7.87 (d, *J*=8.0 Hz, 2H), 7.57 (dd, *J*=2.0, 1.0 Hz, 2H), 7.35 (dd, *J*=8.0, 2.0Hz, 2H). ^{13}C NMR (75 MHz, $CDCl_3$) δ: 140.4, 123.4, 121.9, 121.6, 119.8, 113.9. FT-IR (ATR, cm^{-1}) ν= 3396, 3073, 2920, 2852, 1595, 1420, 1050, 803. HRMS (DART) $C_{12}H_8Br_2N$ m/z calc= 323.90235, found= 323.90222, diff. (ppm)= 0.41

***N*-(4-Iodophenyl)carbazole (11 or 11-*d*$_4$).** Compounds were prepared following a methodology reported and spectroscopic data matched with the previous reports.[3] 0.5 g (3.0 mmol) of carbazole, 1.981 g (6.0 mmol) of 1,4-diiodobenzene, 0.022 g (0.12 mmol) of CuI, 0.015 g (0.06 mmol) of 18-crown-6 and 0.830 g (6.0 mmol) of K_2CO_3 were used to get **11** (yield 74 %). On the other hand, 0.5 g (3.0 mmol) of carbazole, 2.0 g (6.0 mmol) of 1,4-diiodobenzene-*d*$_4$, 0.023 g (0.12 mmol) of CuI, 0.016 g (0.06 mmol) of 18-crown-6 and 0.831 g (6.0 mmol) of K_2CO_3 were used to give **11-*d*$_4$** (yield 78 %).

X-Ray diffraction studies

Data collection were performed at 298 K on a Bruker-APEX-II CCD diffractometer with Mo Kα-radiation, λ = 0.71073 Å. The structures were solved by direct methods and refined using SHELXL-2014. All non-hydrogen atoms were refined anisotropically.

Crystallization of title compounds

Prisms of rotor **1** were obtained from slow evaporation of a dichloromethane solution at room temperature. The single crystal X-ray data was solved and refined in an orthorhombic space

group *P*bca with eight molecules in the unit cell (*Z*=8). Complementarily, thin colorless prisms of molecular rotor **2** were obtained from slow evaporation of an *N,N*-dimethylformamide solution at room temperature. The X-ray diffraction data was solved and refined in a monoclinic space group *C*2/c with *Z*=4. Single crystals of **3** suitable for X-ray diffraction were obtained from a THF solution by slow evaporation at room. They were solved and refined in the monoclinic system, space group $P2_1$/c with *Z*=2. Finally, single crystals of compound **4** were grown by evaporation of a dichloromethane solution at room temperature, solved in the monoclinic system with a $C2_1$/c space group.

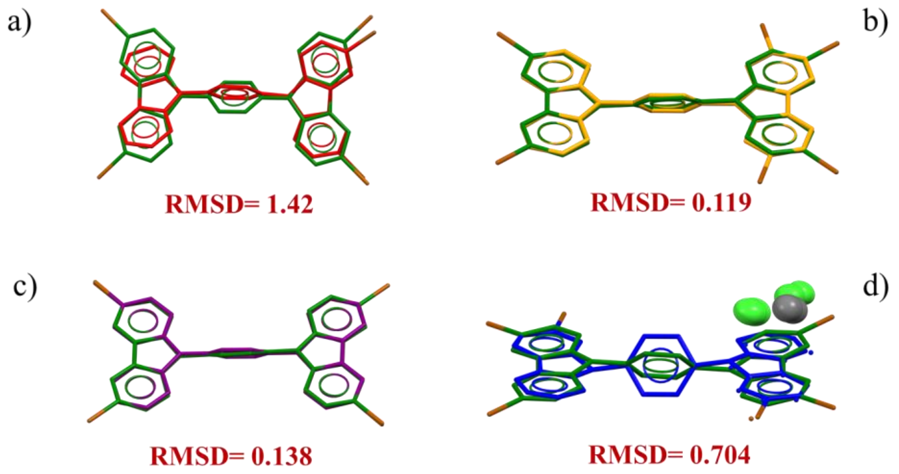

Figure S1. *Structure overlay of title compounds with amphidynamic crystal 3,6-tetrabromo-bis(carbazoyl)phenylene[4] showing their Root Mean Square Distance (RMSD). a) Compound* ***1*** *b) Compound* ***2*** *c) Compound* ***3*** *and d) Compound* ***4***

Compound 1

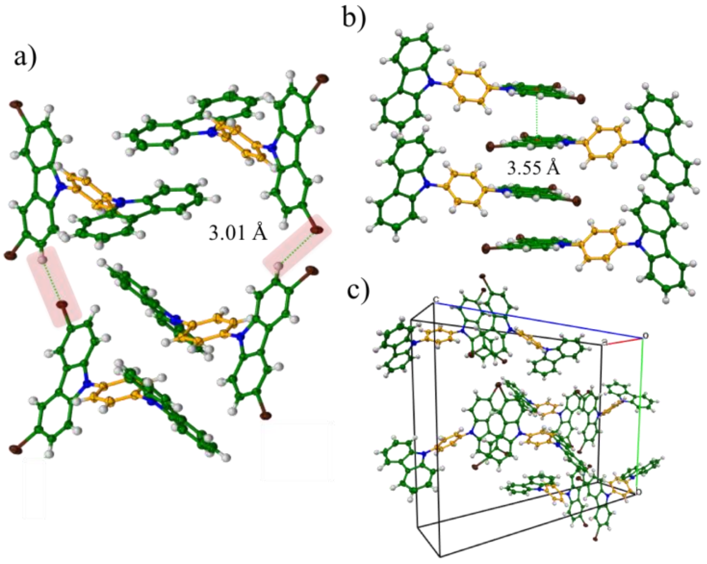

Figure S2. *Compound **1** a) Non-classical hydrogen bond CH···Br b) π-stacking c) unit cell*

Compound 2

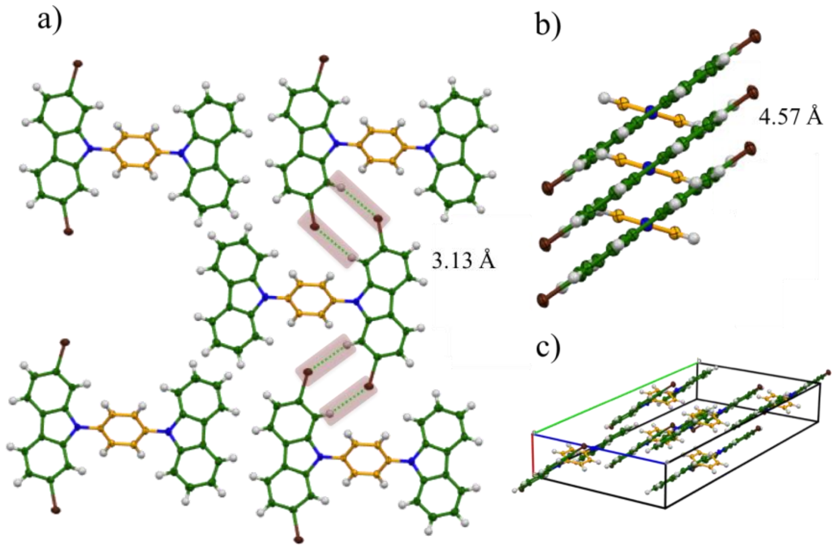

Figure S3. *Crystalline array in compound **2** a) CH···Br interactions b) π-stacking c) unit cell*

Compound 3

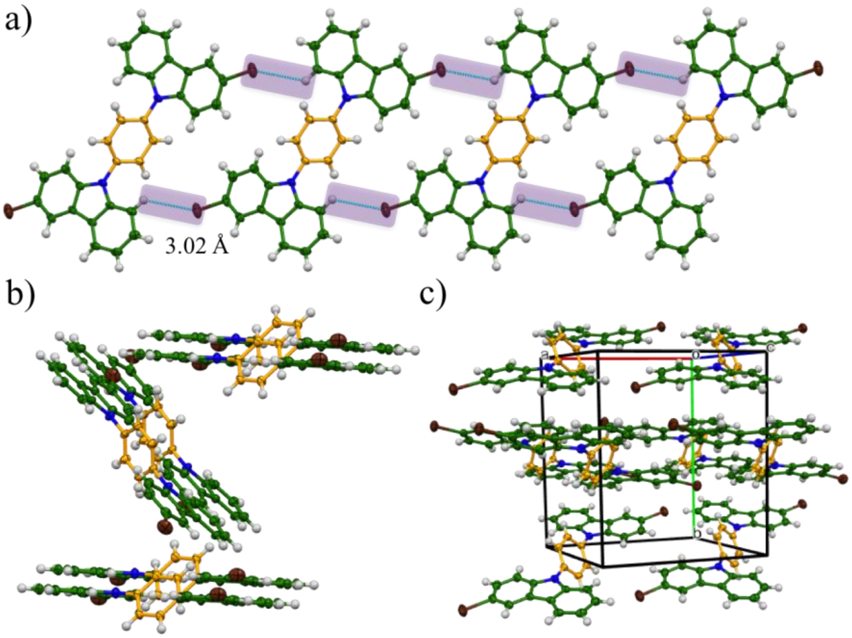

Figure S4. *Crystal structure of compound* ***3*** *a) CH···Br interactions b) zig-zag arrangement along c axe and c) unit cell*

Compound 4

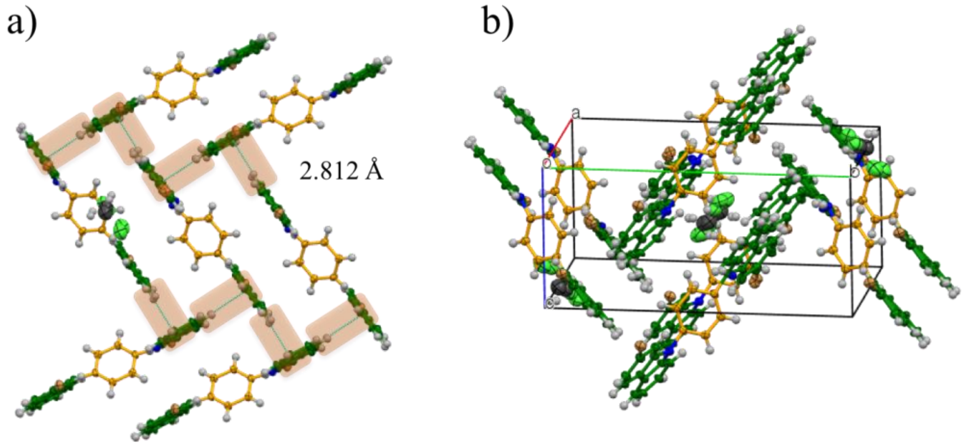

Figure S5. *Compound* ***4*** *a) C-H···π interactions in crystalline packing and b) unit cell with DCM molecules included*

Compound 4 solvent-free

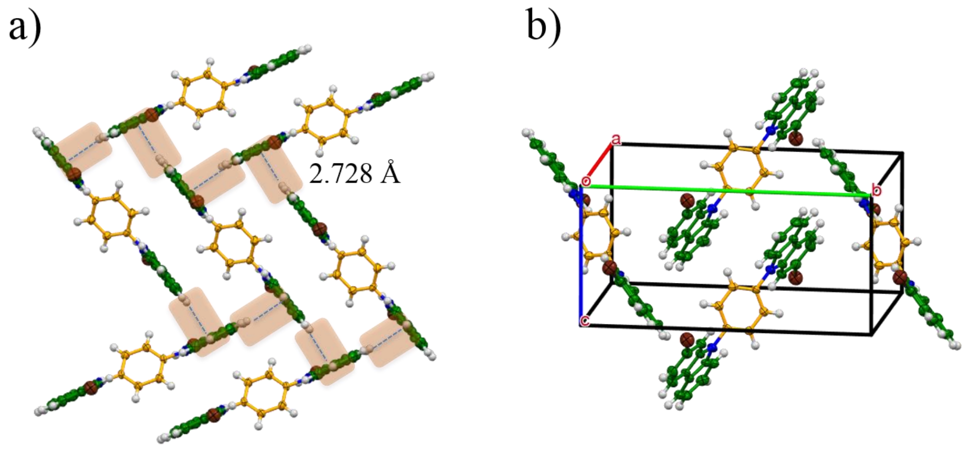

Figure S6. *Compound* ***4 solvent free*** *structure a) C-H···π interactions in crystalline packing and b) unit cell without solvent molecules*

Table S1. Crystallographic and refinement data of compounds **1-4**

Compound	***1***	***2***	***3***	***4***	***4 (solvent free)***
formula	$C_{30}H_{18}Br_2N_2$	$C_{30}H_{18}Br_2N_2$	$C_{30}H_{18}Br_2N_2$	$C_{62}H_{40}Br_4Cl_4N_4$	$C_{30}H_{18}Br_2N_2$
formula weight	566.26	566.28	566.26	1302.42	566.28
temperature/K	298	298	298	298	298
crystal system	orthorhombic	monoclinic	monoclinic	monoclinic	monoclinic
space group	*P*bca	*C*2/c	$P2_1$/c	$P2_1$/c	$P2_1$/c
a/Å	7.0412(7)	4.5701(2)	11.1353(5)	9.2687(9)	9.2718(16)
b/Å	22.373(2)	30.1408(16)	13.5027(7)	17.9615(19)	17.063(3)
c/Å	29.798(3)	16.5121(8)	8.2770(4)	8.2589(9)	8.0988(14)
α/°	90	90	90	90	90
β/°	90	92.410(1)	109.689(1)	94.185(3)	96.262(5)
γ/°	90	90	90	90	90
volume/$Å^3$	4694.2(8)	2272.47(19)	1171.74(10)	1371.3(2)	1273.6(4)
Z	8	4	2	1	2
ρcalc g/cm^3	1.602	1.655	1.605	1.577	1.477
μ/mm^{-1}	3.476	3.590	3.481	3.174	3.203
F(000)	2256	1128	564	648	564
crystal size	0.05x0.18x0.40	0.04x0.19x0.51	0.19x0.27x0.46	0.41x0.34x0.18	0.026x0.142x0.189
radiation/Å	MoKa 0.71073	MoKa 0.71073	MoKa 0.71073	MoKa 0.71073	MoKa 0.71073
2θ range	2.3 to 25.4	2.7 to 30.0	2.5 to 29.6	2.7 to 26.4	2.21 to 29.12
index ranges	-8≤h≤8<br>-27≤k≤26<br>-35≤l≤35	-6≤h≤6<br>-42≤k≤42<br>-23≤l≤23	-15≤h≤15<br>-18≤k≤18<br>-11≤l≤10	-11≤h≤10<br>-22≤k≤22<br>-10≤l≤10	-12≤h≤12<br>-23≤k≤23<br>-11≤l≤11
reflns collected	28240	22659	34885	8516	39214
Independent reflns	4326	3338	3287	3495	3424
reflns observed [*I*≥2σ(*I*)]	2470	2112	1900	2805	1652
GOF on F^2	1.05	1.02	1.02	1.05	1.01
Largest diff. peak e/$Å^3$	-0.46/0.55	-0.26/0.45	-0.38/0.42	-0.40/1.52	-0.41/0.55
final R indexes [*I*≥2(*I*)]	R=0.07<br>wR2= 0.12	R=0.04<br>wR2= 0.0971	R=0.05<br>wR2= 0.1306	R=0.06<br>wR2= 0.1989	R=0.06<br>wR2= 0.1338

Solid state ^{2}H NMR quadrupolar echo spin

Solid state ^{2}H NMR experiments were carried out on a Bruker AV300 spectrometer operating at a frequency of 46.07 MHz using a 4 mm wide-line probe with a π/2 pulse of 2.5 μs and recycle delay of 20 s (compounds **1** and **3**) and a spectrometer in which the ^{2}H nucleous resonates at 92.1 MHz with a 5 mm wide-line probe and a π/2 pulse of 2.9 μs were used (compound ***2***). The spectra were acquired by averaging at least 256 scans and processed with a line broadening of 2 kHz. The sample was placed inside a borosilicate glass NMR tube between two glass rods. The temperature inside the probe was calibrated by using the shift of ^{207}Pb as the reference. The ^{2}H NMR line shapes were simulated using the NMRWebLab 6.0.4; in all cases a Quadrupolar Coupling Constant (QCC) of 170 kHz was employed.

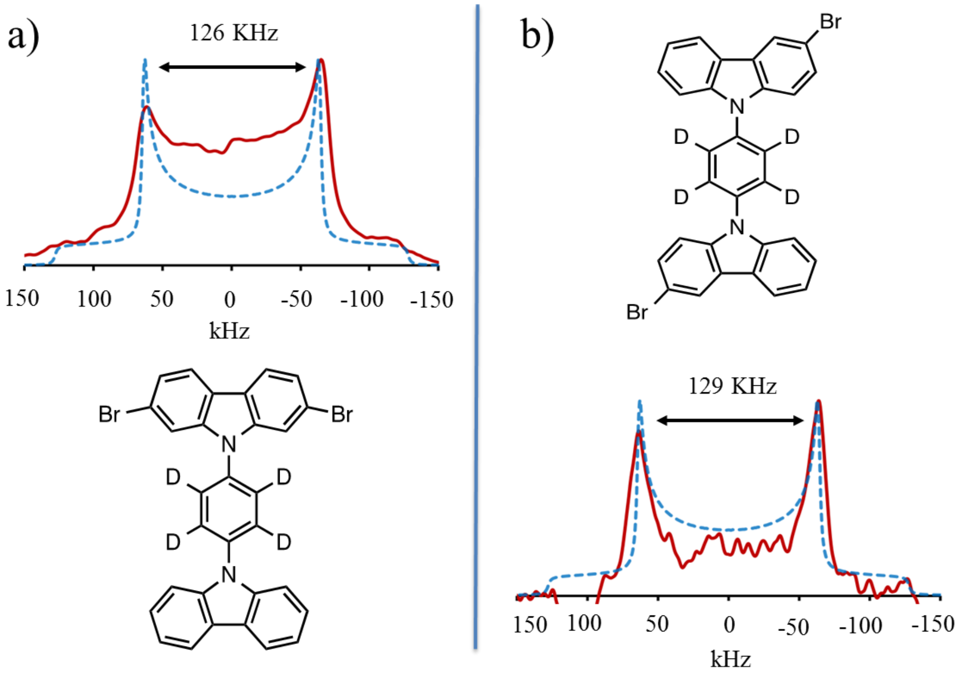

Figure S7. *^{2}H NMR quadrupolar echo experiments of title compounds at high temperature: experimental (solid line) and calculated (dotted line) a) Compound **2** (372 K) and b) Compound **3** (373 K)*

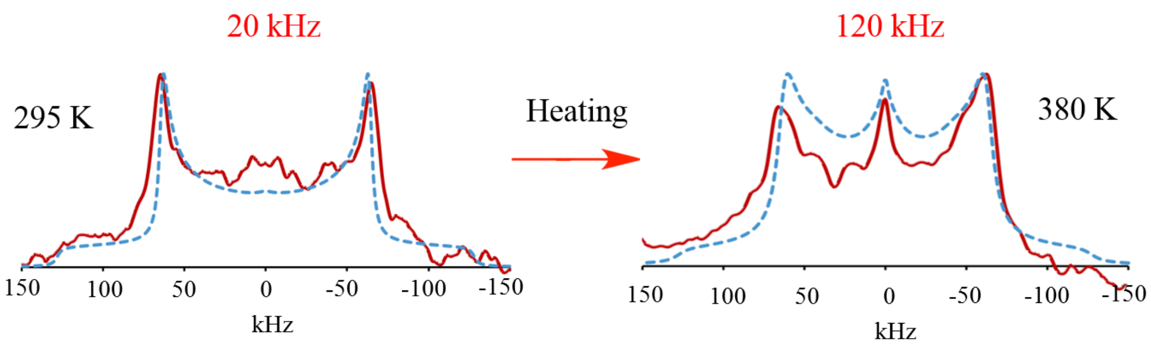

Figure S8. *^{2}H NMR simulated line shapes for compound* ***1*** *using a QCC of 170 kHz, cone angle of 54.7º, angular displacement of 90º (4-fold), pulse delay of 50 μs and populations of 0.45:0.05:0.45:0.05*

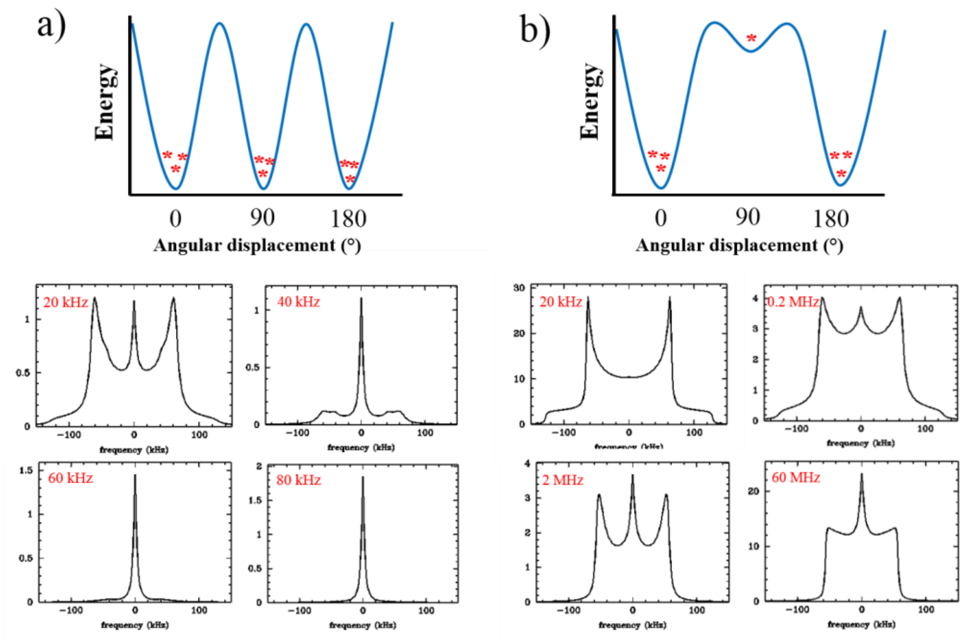

Figure S9. *^{2}H NMR simulated line shapes using a QCC of 170 kHz, cone angle of 54.7º, angular displacement of 90º (4-fold) and pulse delay of 50 μs with different rotation frequencies showing the evolution of the extremely narrow signal. a) Symmetrical potential with populations 0.25:0.25:0.25:0.25 and b) Asymmetrical potential with populations 0.47:0.03:0.47:0.03*

^{1}H and ^{13}C Nuclear Magnetic Resonance spectra in solution

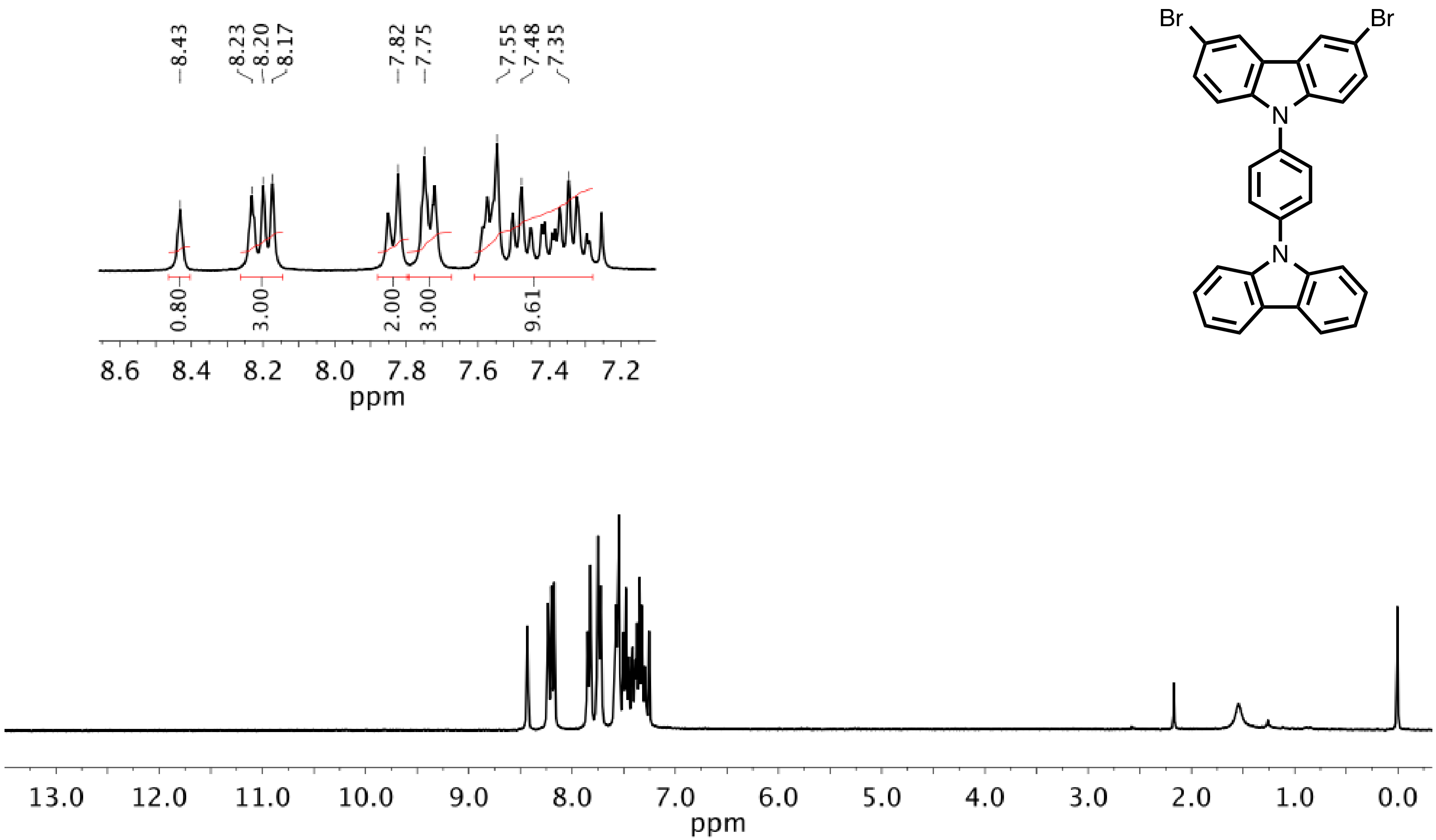

Figure S10. Spectrum of compound **1** (300 MHz, $CDCl_3$)

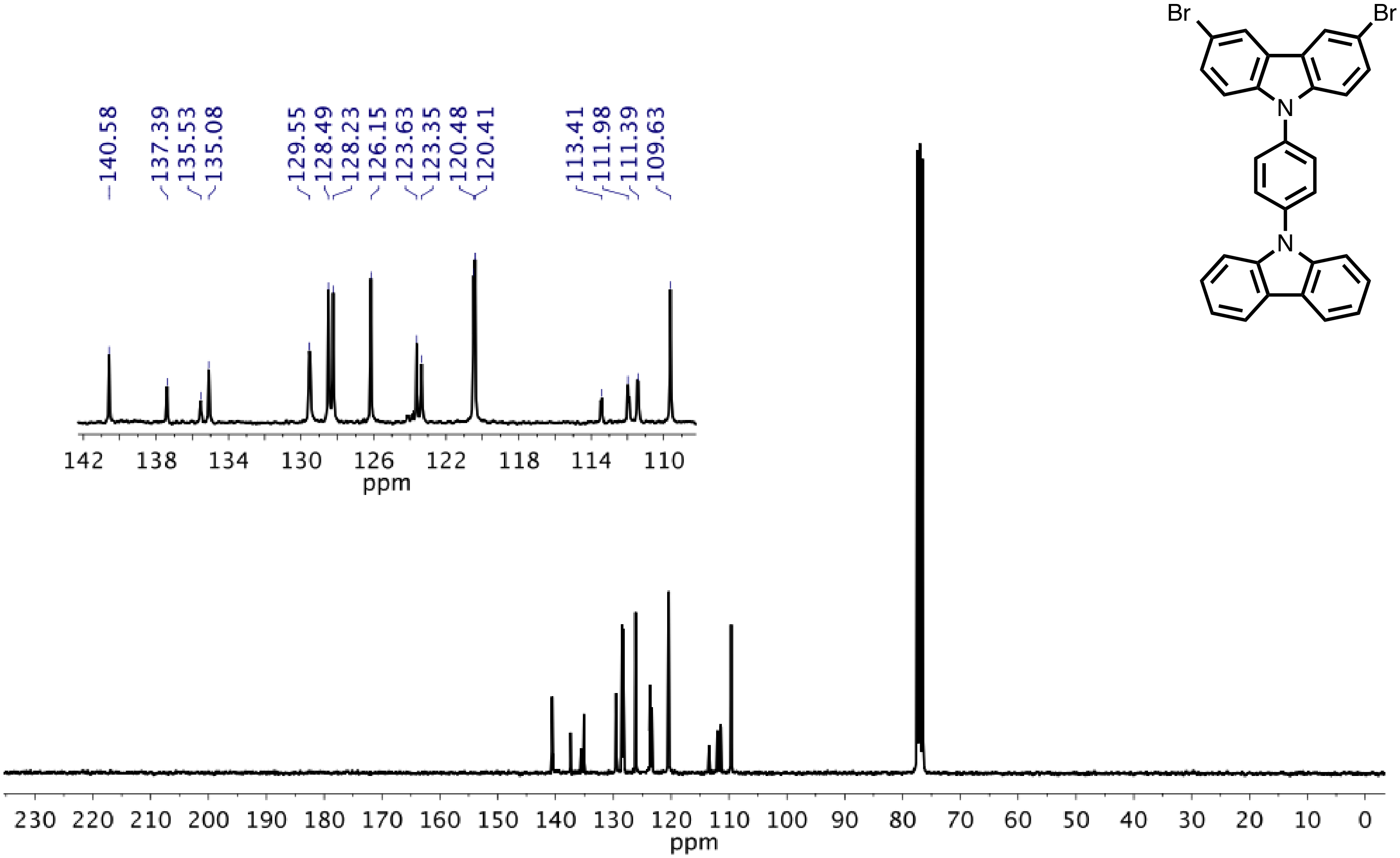

Figure S11. Spectrum of compound **1** (75 MHz, $CDCl_3$)

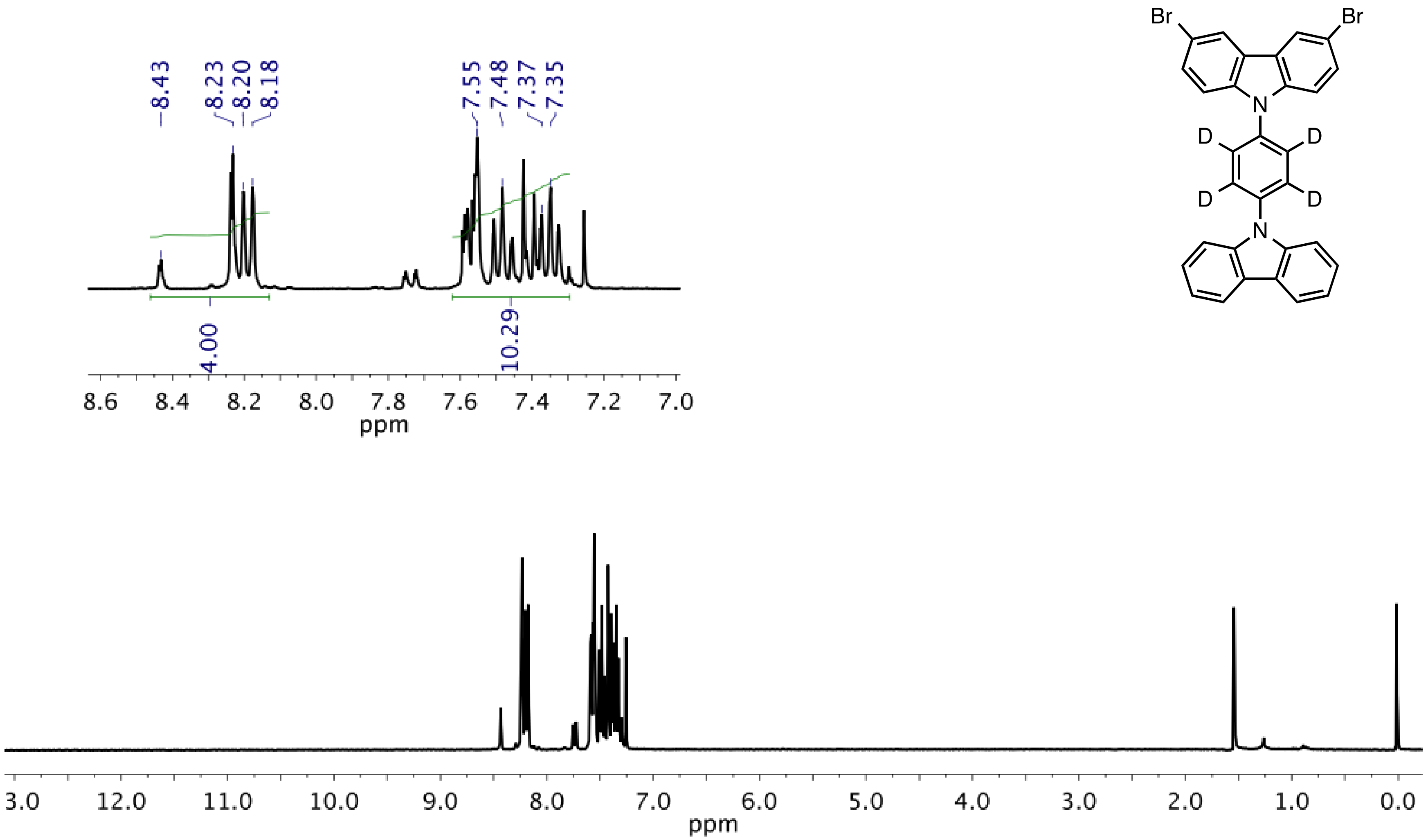

Figure S12. Spectrum of compound **1-*d*$_4$** (300 MHz, $CDCl_3$)

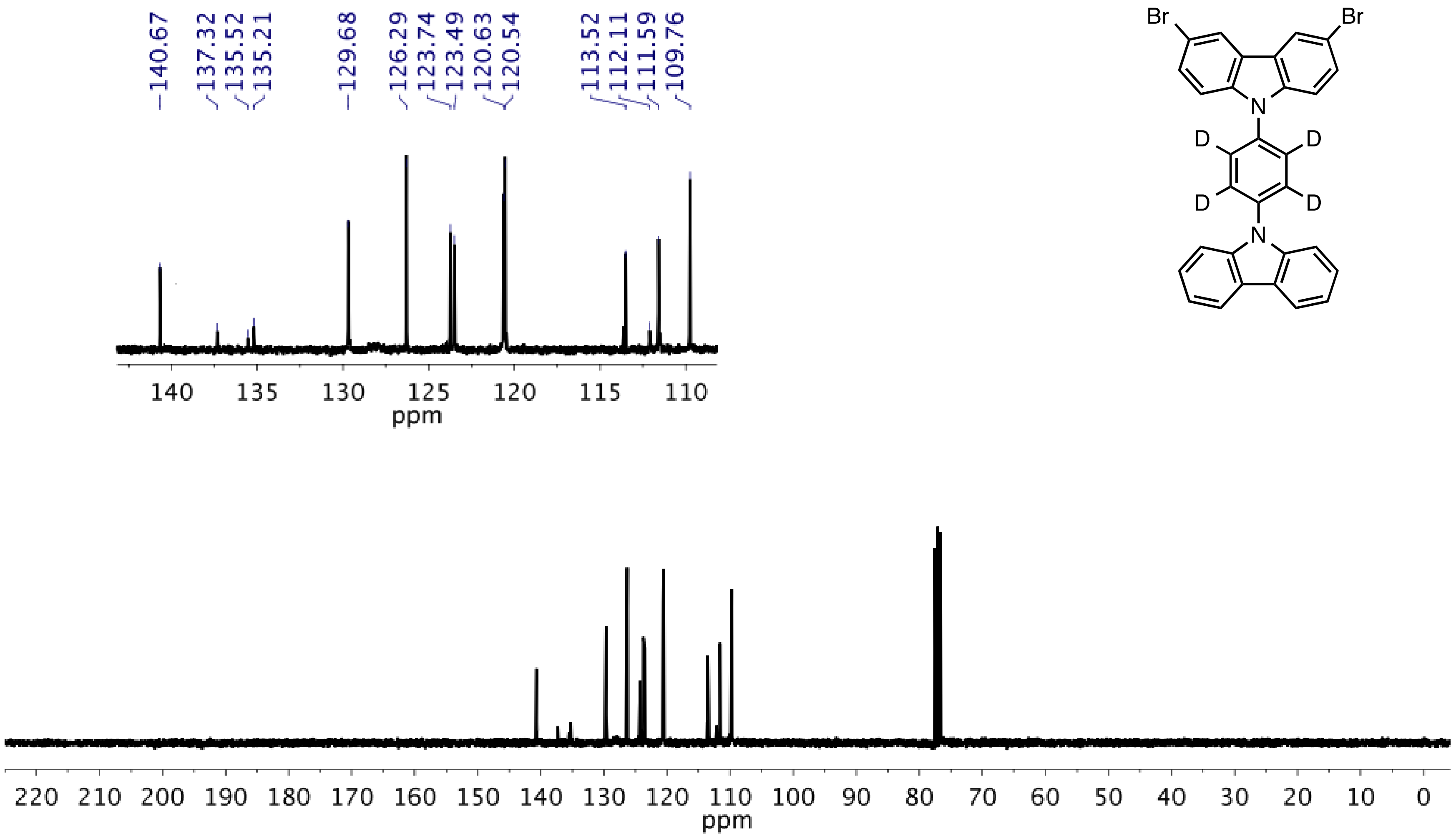

Figure S13. Spectrum of compound **1-*d*₄** (75 MHz, $CDCl_3$)

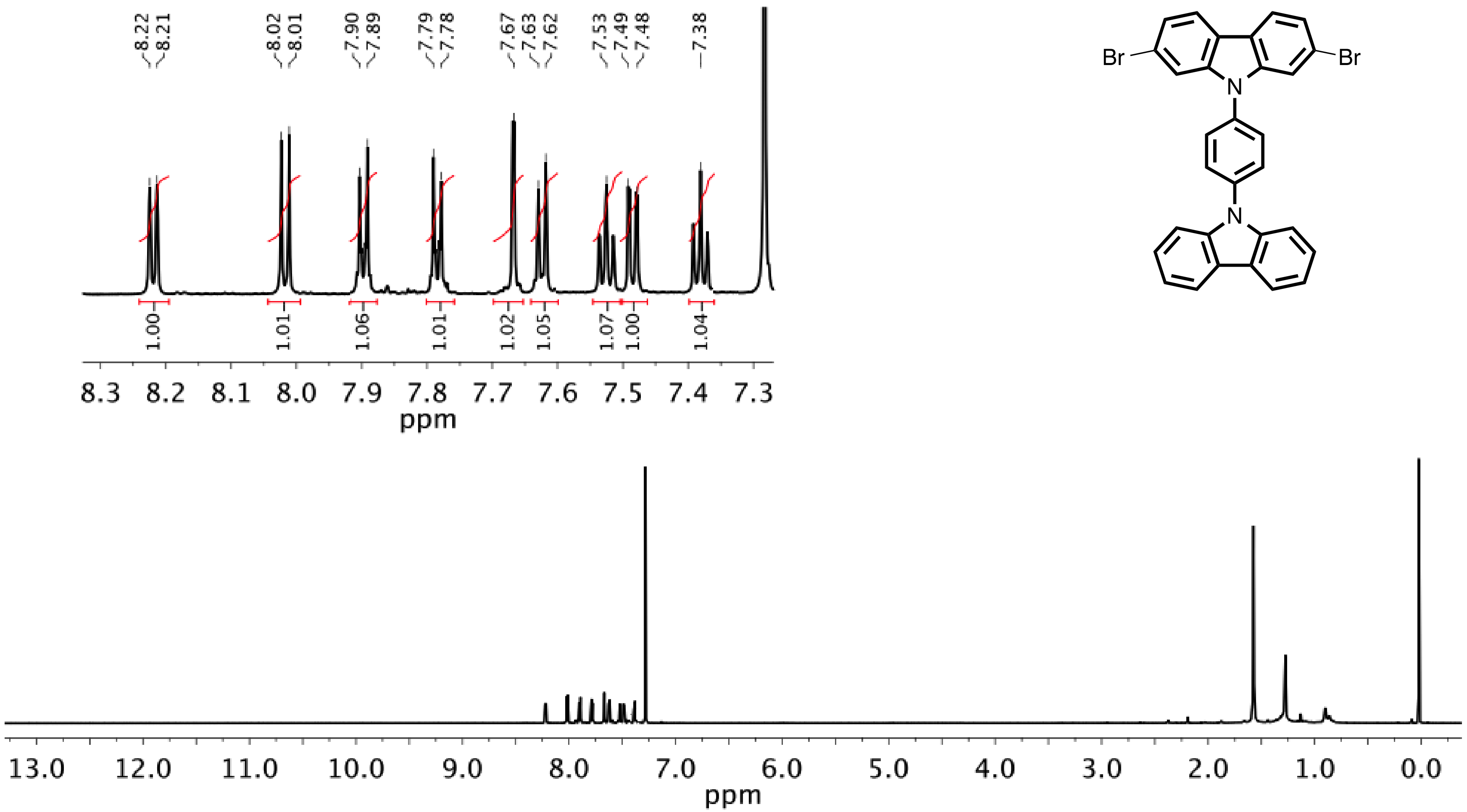

Figure S14. Spectrum of compound **2** (700 MHz, $CDCl_3$)

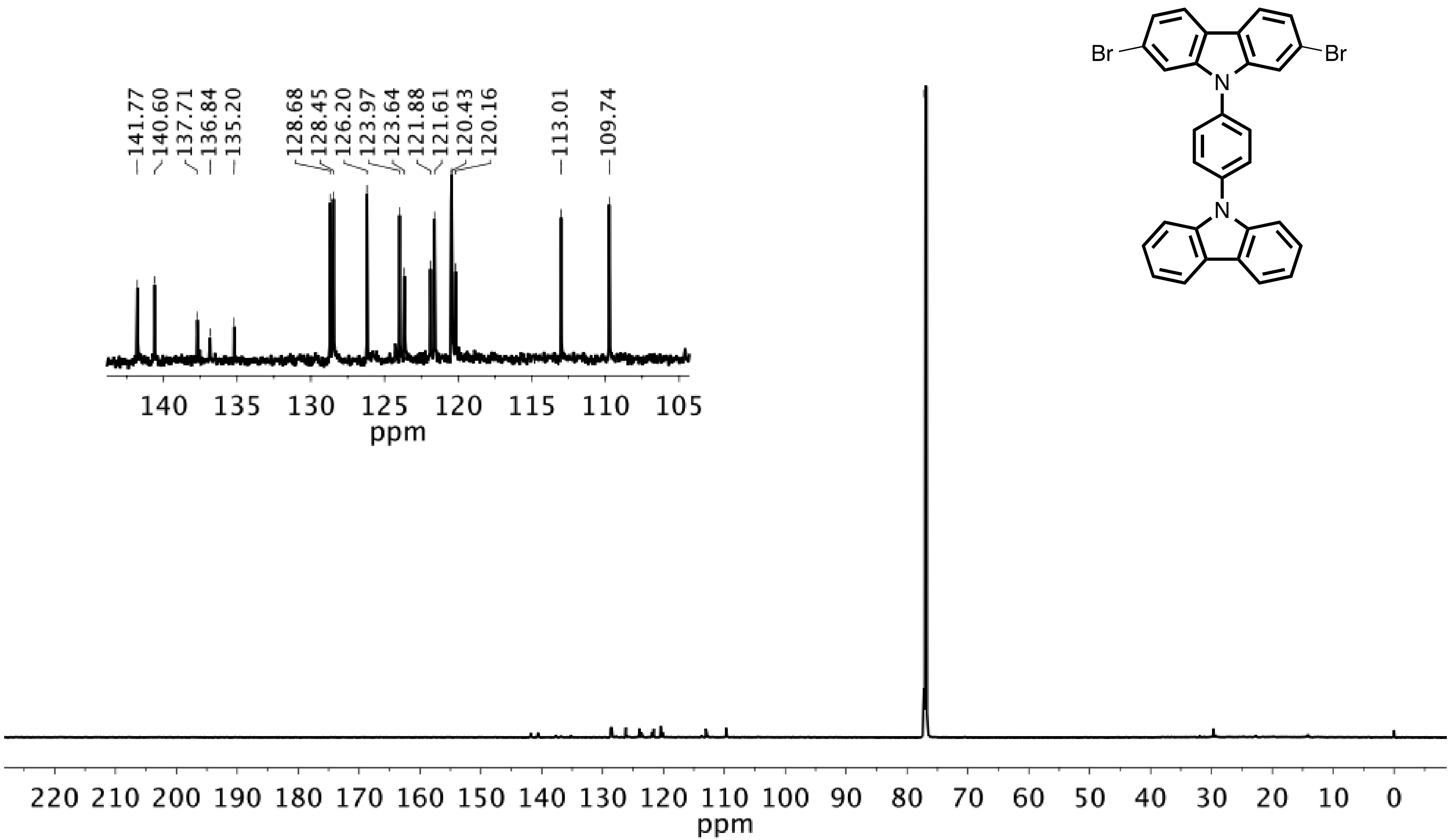

Figure S15. Spectrum of compound **2** (175 MHz, $CDCl_3$)

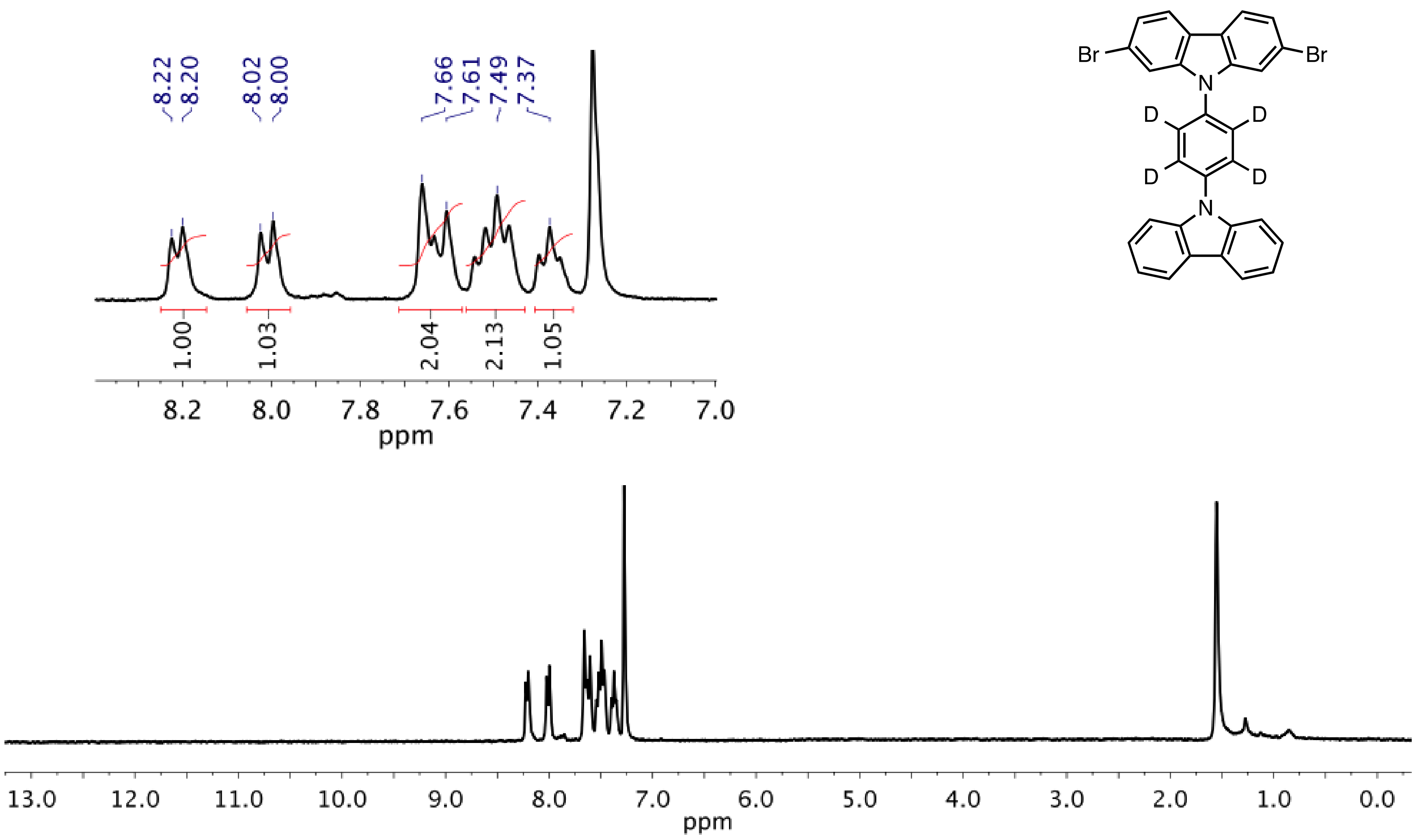

Figure S16. Spectrum of compound **2-*d*4** (300 MHz, $CDCl_3$)

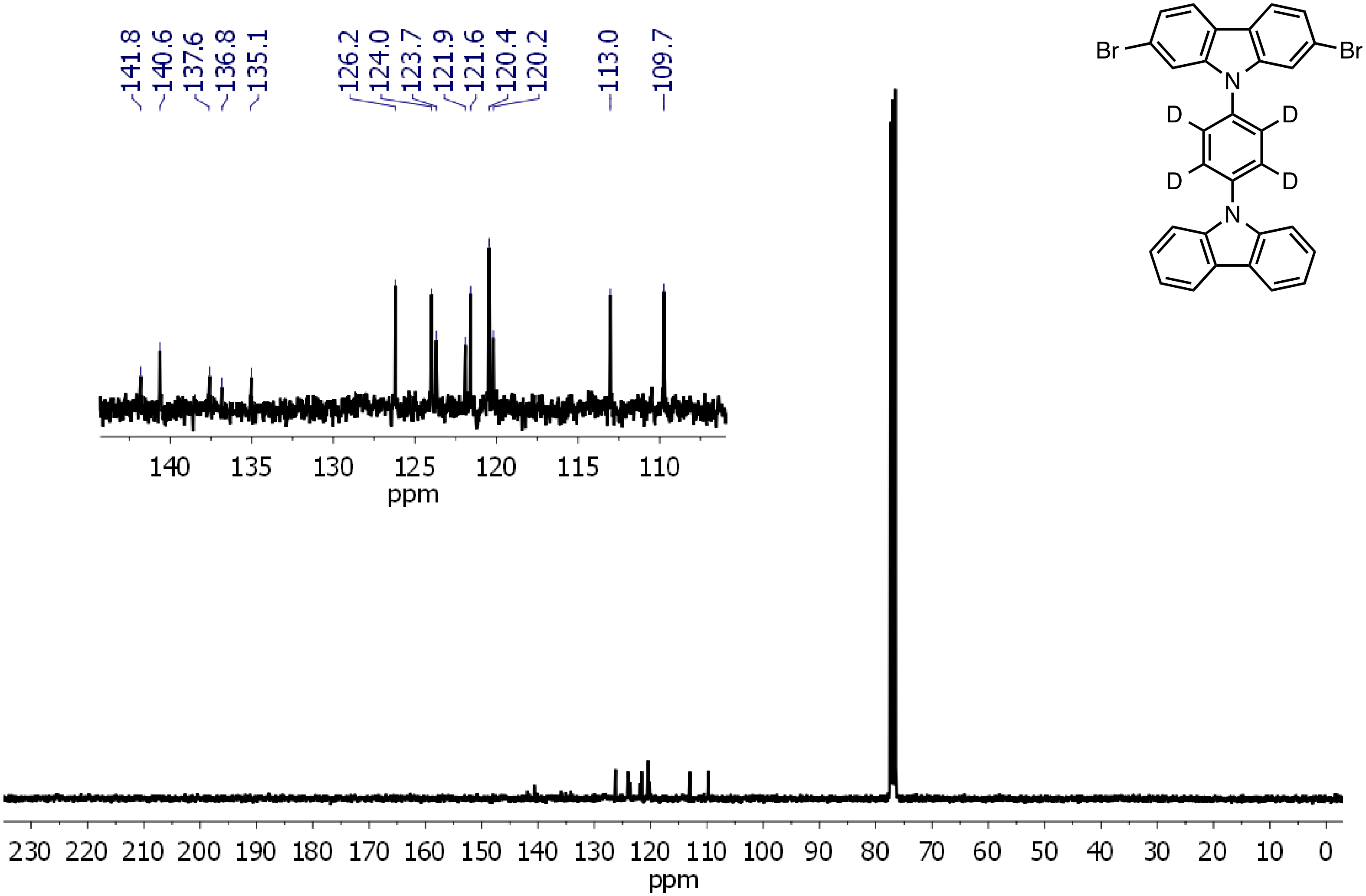

Figure S17. Spectrum of compound **2-*d*4** (75 MHz, $CDCl_3$)

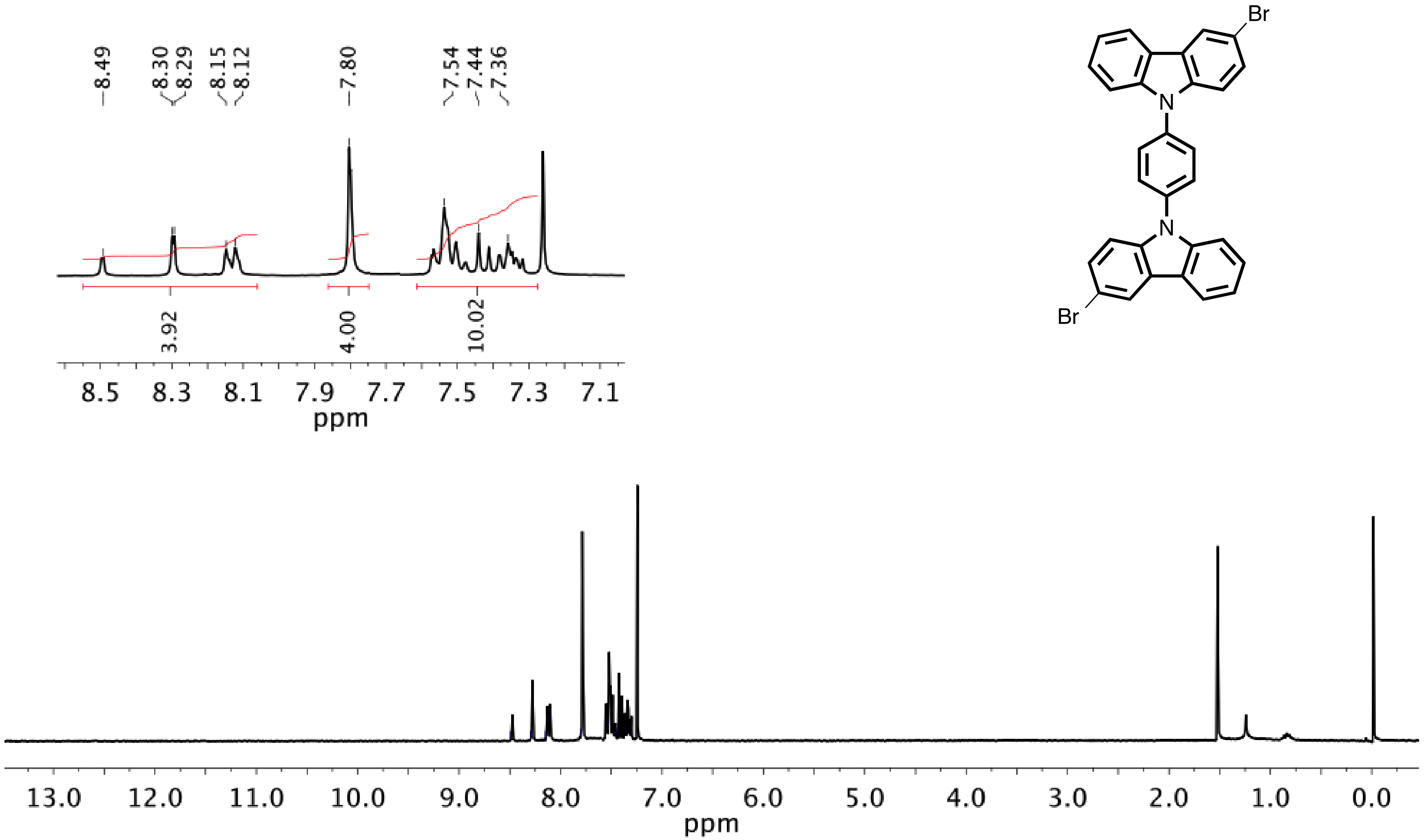

Figure S18. Spectrum of compound **3** (300 MHz, $CDCl_3$)

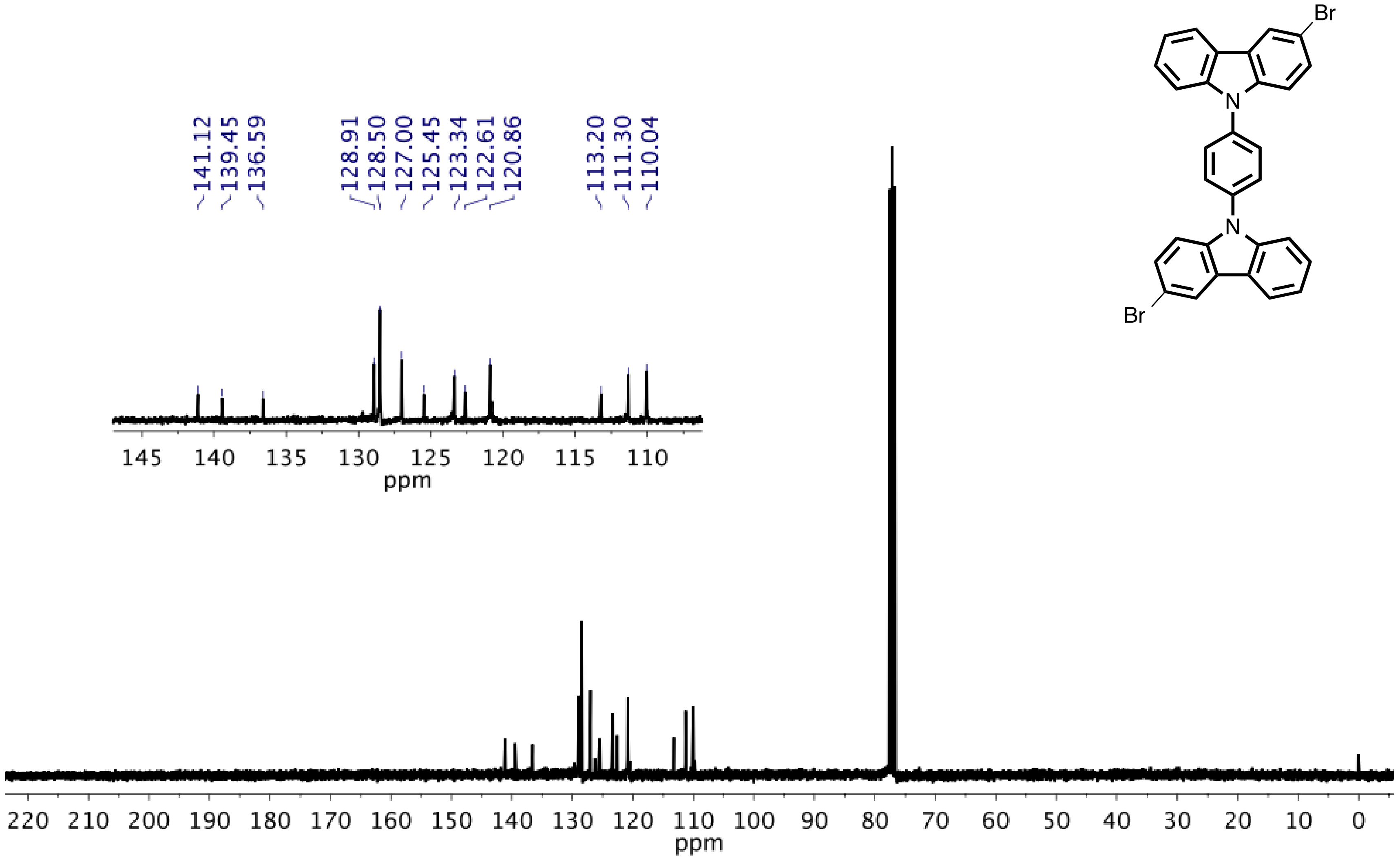

Figure S19. Spectrum of compound **3** (75 MHz, $CDCl_3$)

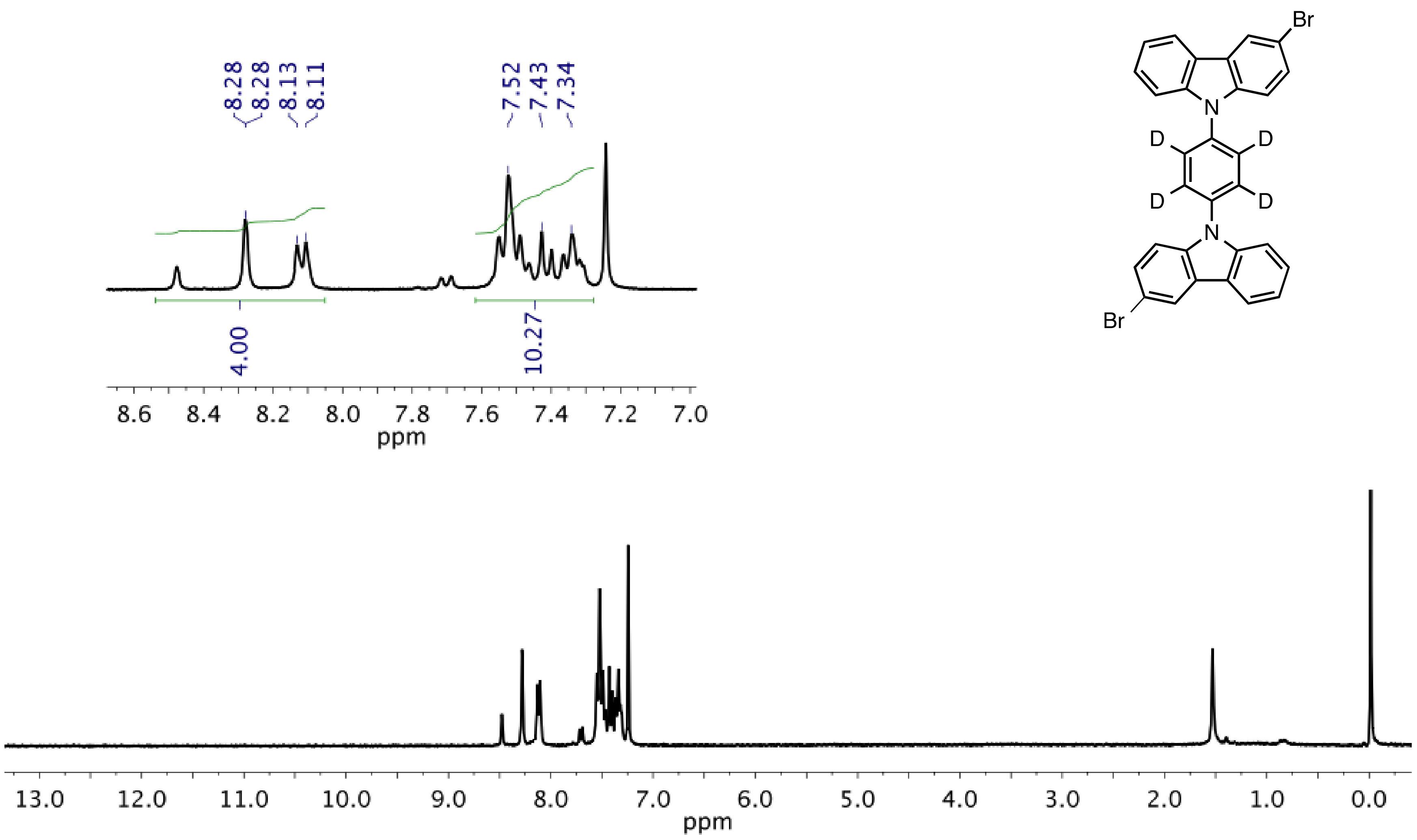

Figure S20. Spectrum of compound **3-*d*$_4$** (300 MHz, $CDCl_3$)

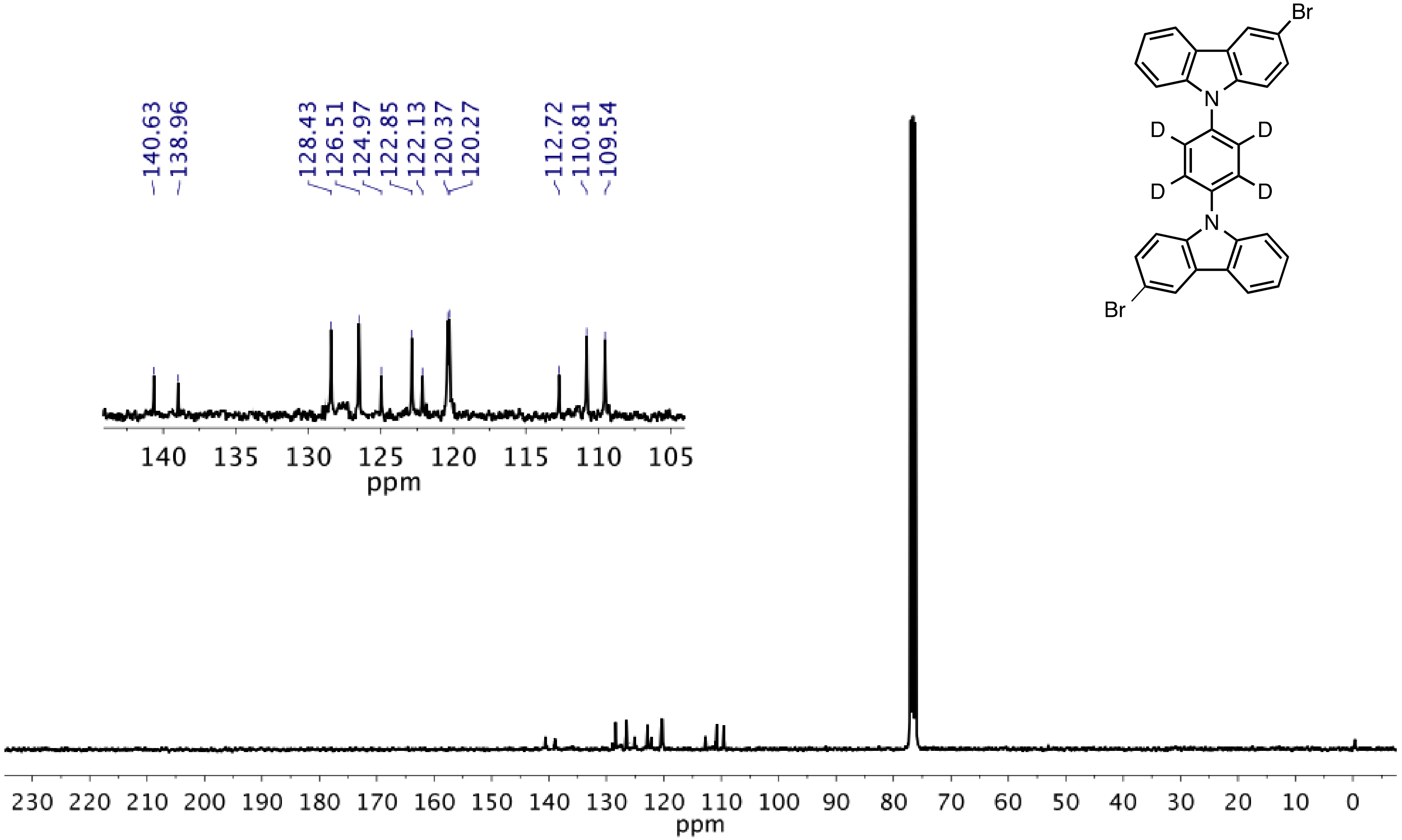

Figure S21. Spectrum of compound **3-*d*₄** (75 MHz, $CDCl_3$)

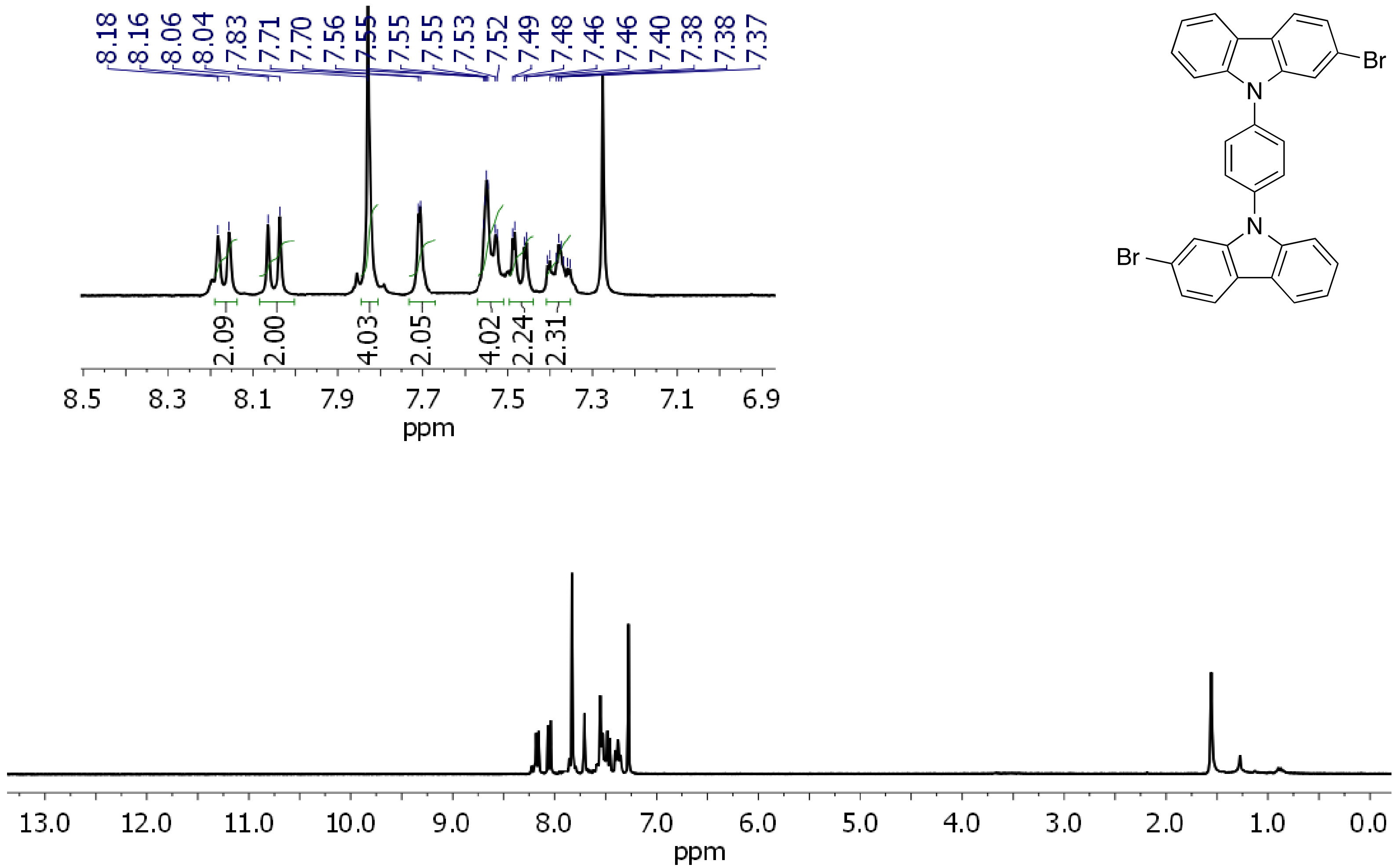

Figure S22. Spectrum of compound **4** (300 MHz, $CDCl_3$)

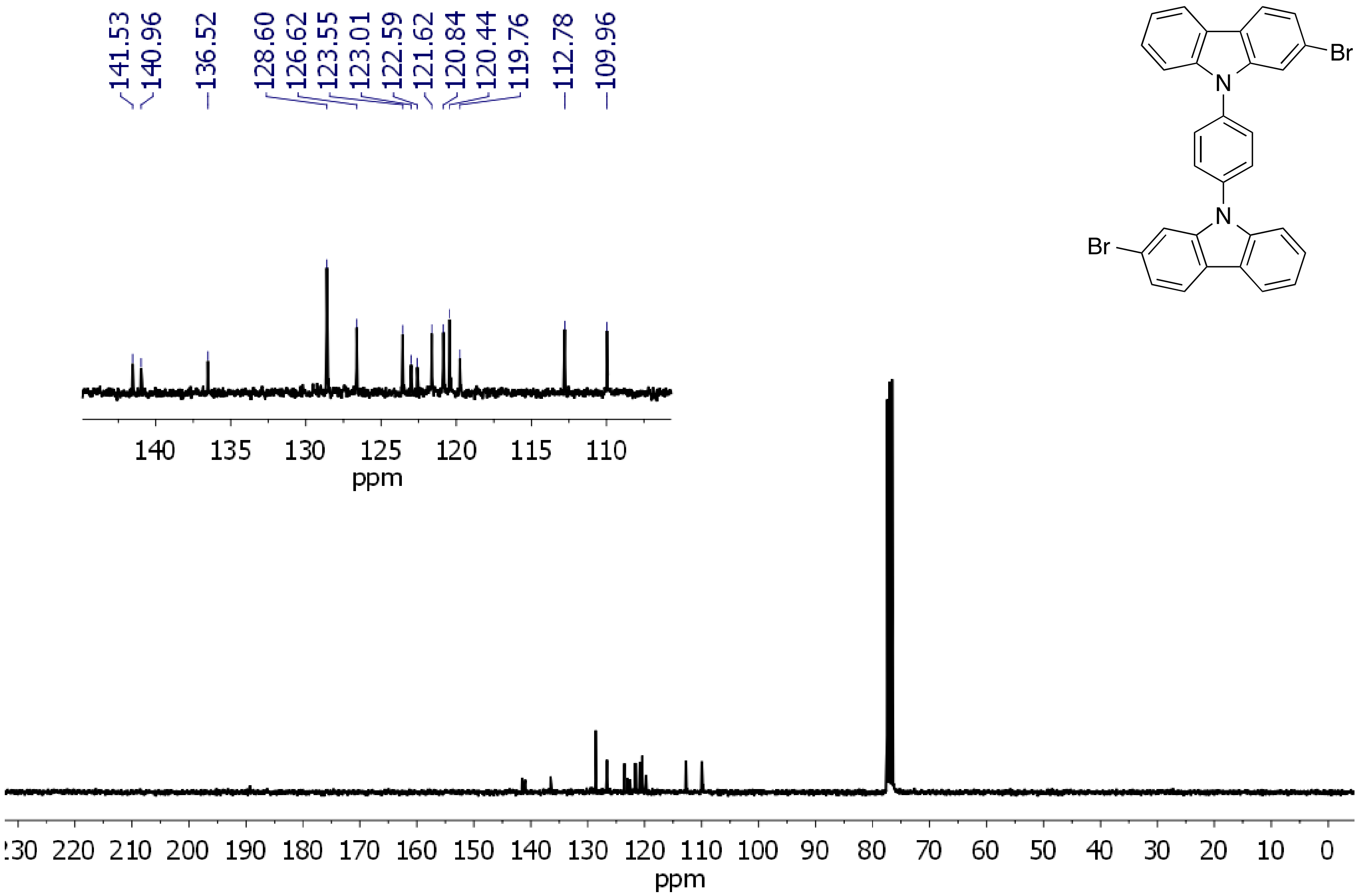

Figure S23. Spectrum of compound **4** (75 MHz, $CDCl_3$)

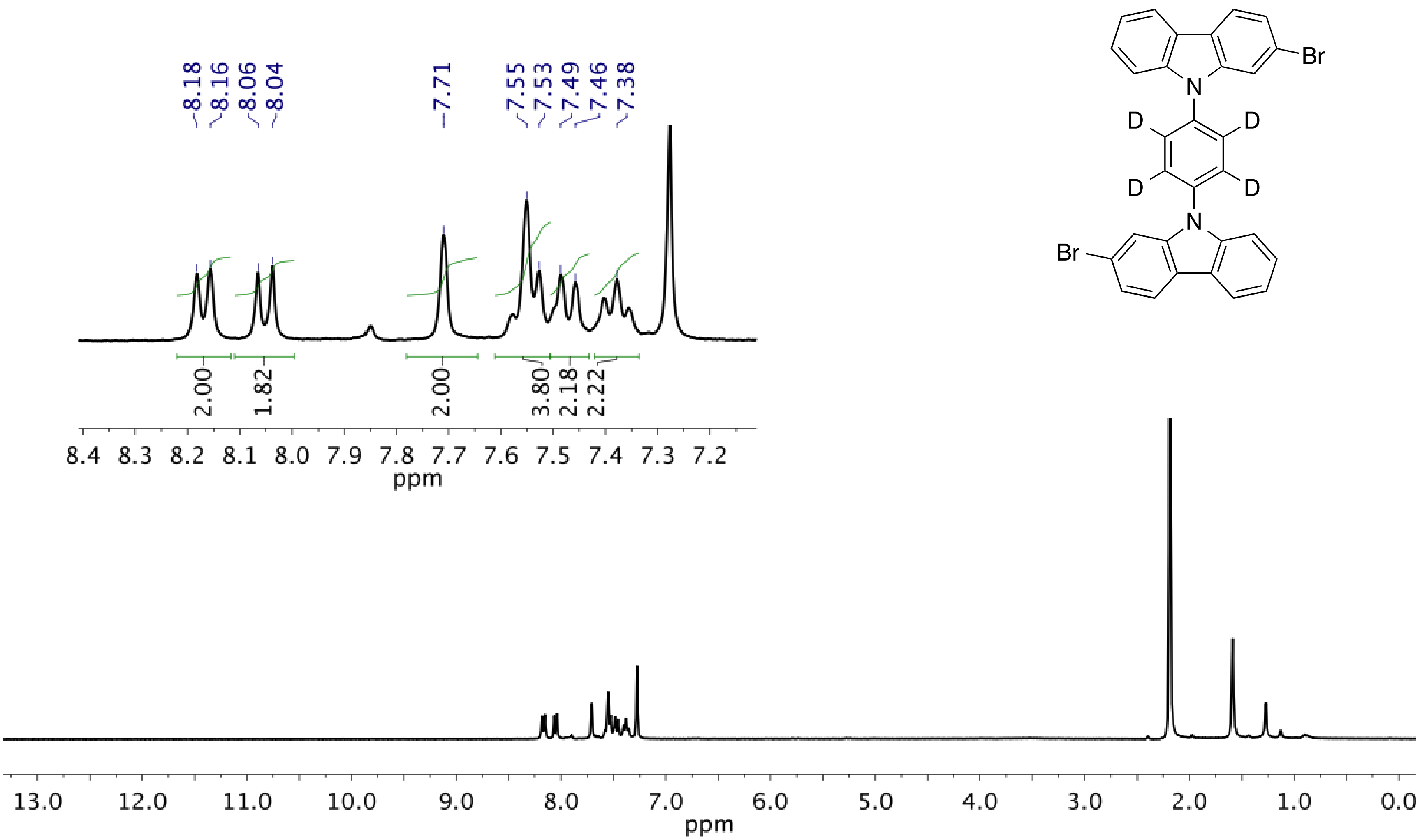

Figure S24. Spectrum of compound ***4-d₄*** (300 MHz, $CDCl_3$)

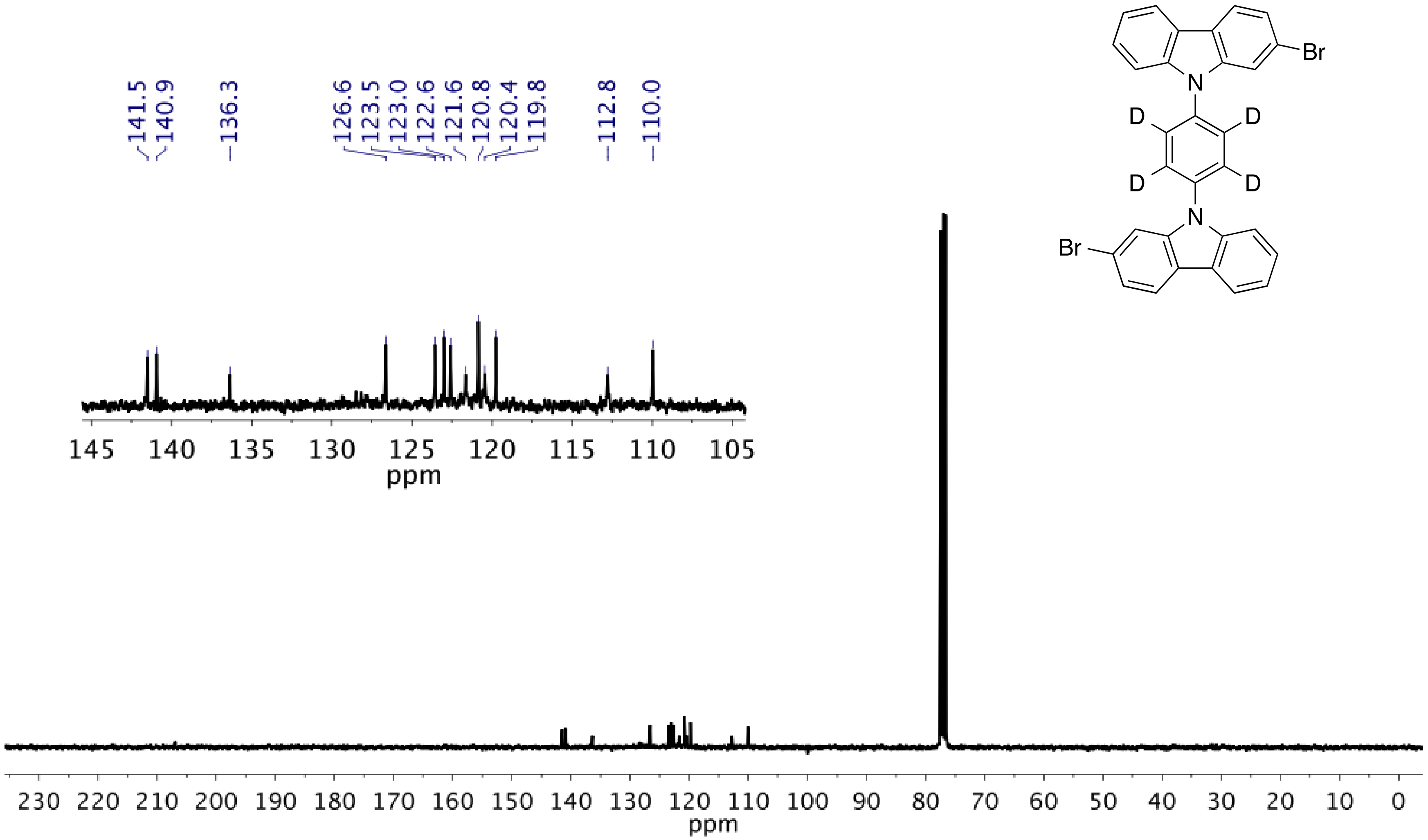

Figure S25. Spectrum of compound **4-*d*₄** (75 MHz, $CDCl_3$)

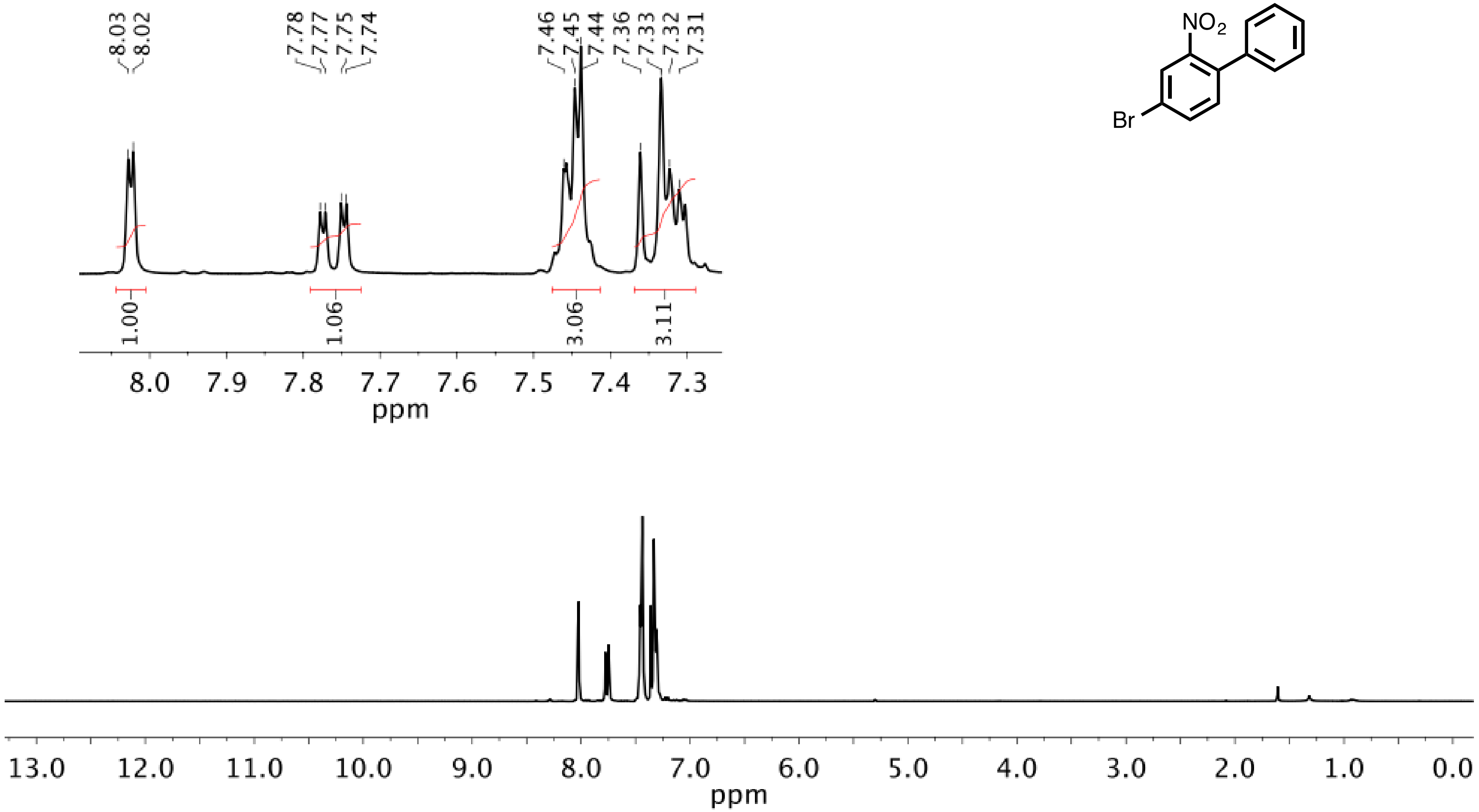

Figure S26. Spectrum of compound **8** (300 MHz, $CDCl_3$)

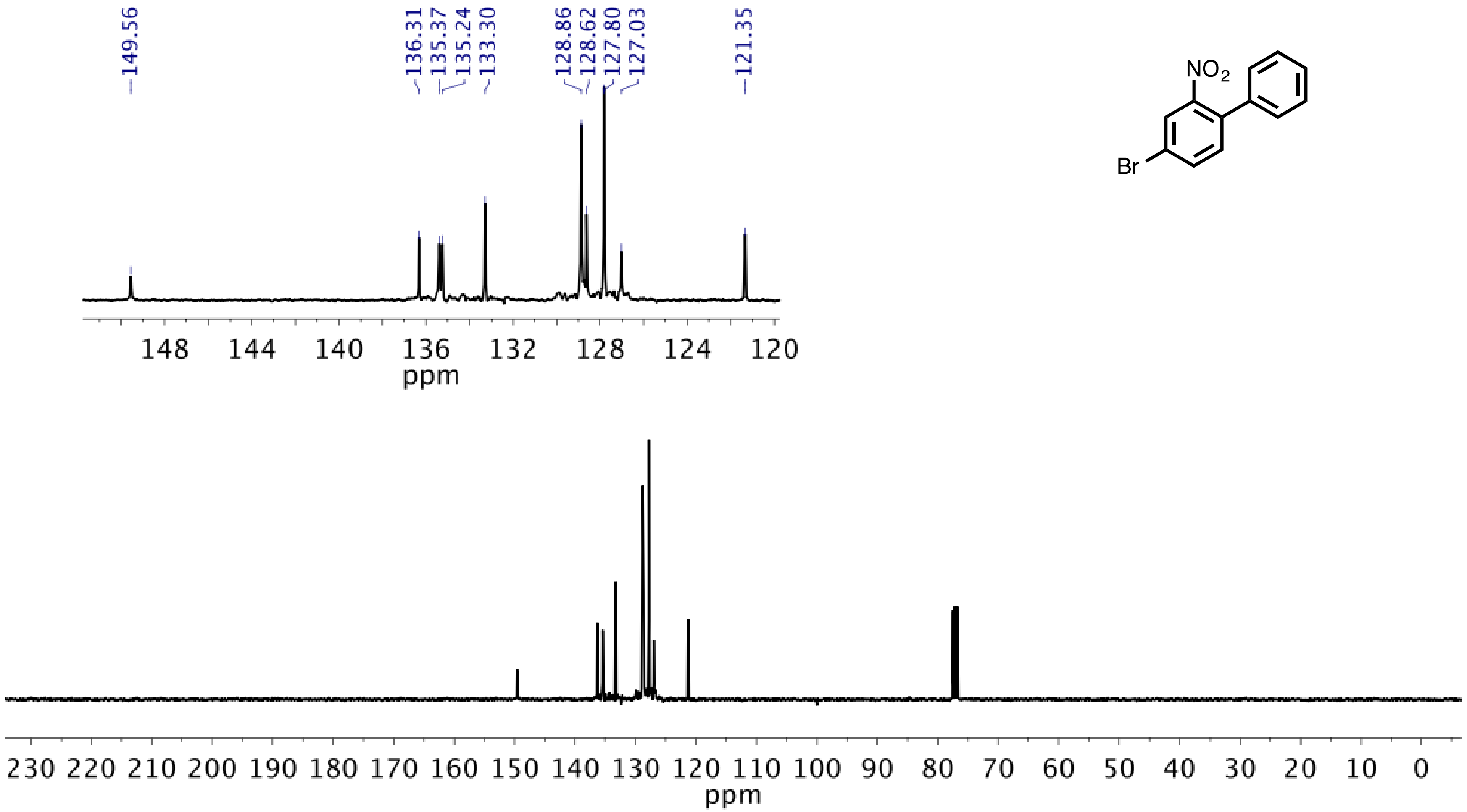

Figure S27. Spectrum of compound **8** (75 MHz, $CDCl_3$)

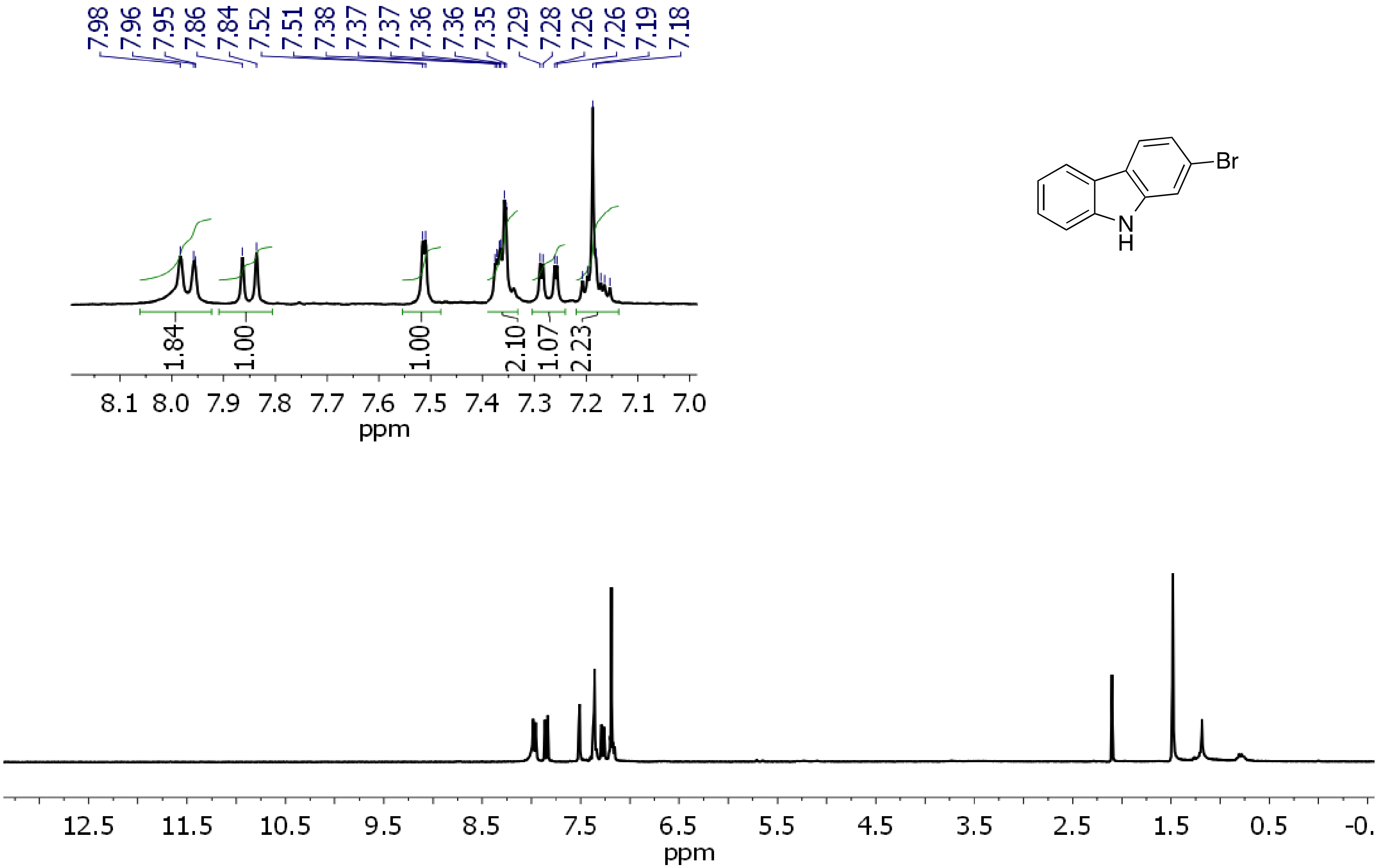

Figure S28. Spectrum of compound **7** (300 MHz, $CDCl_3$)

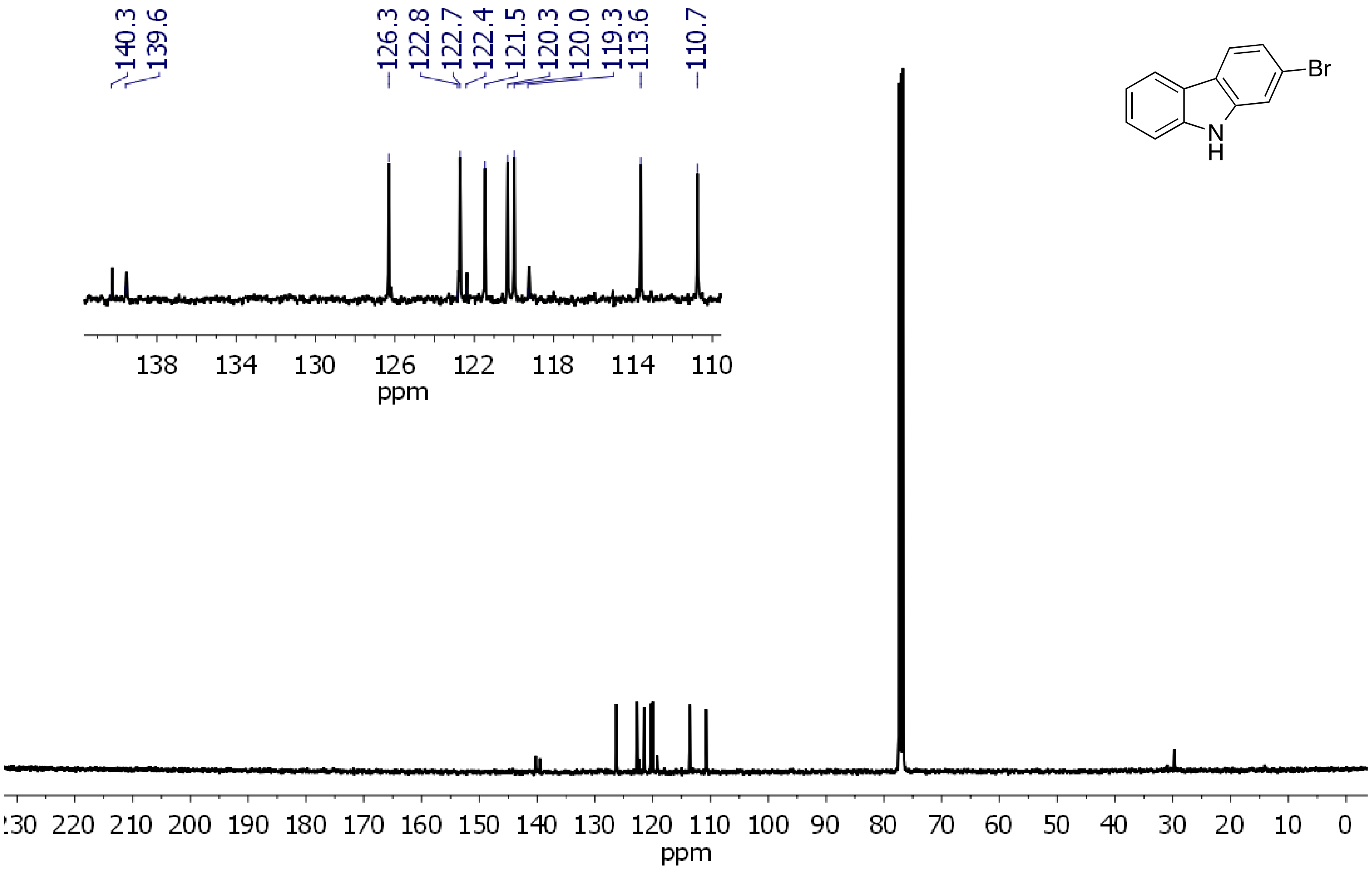

Figure S29. Spectrum of compound **7** (75 MHz, $CDCl_3$)

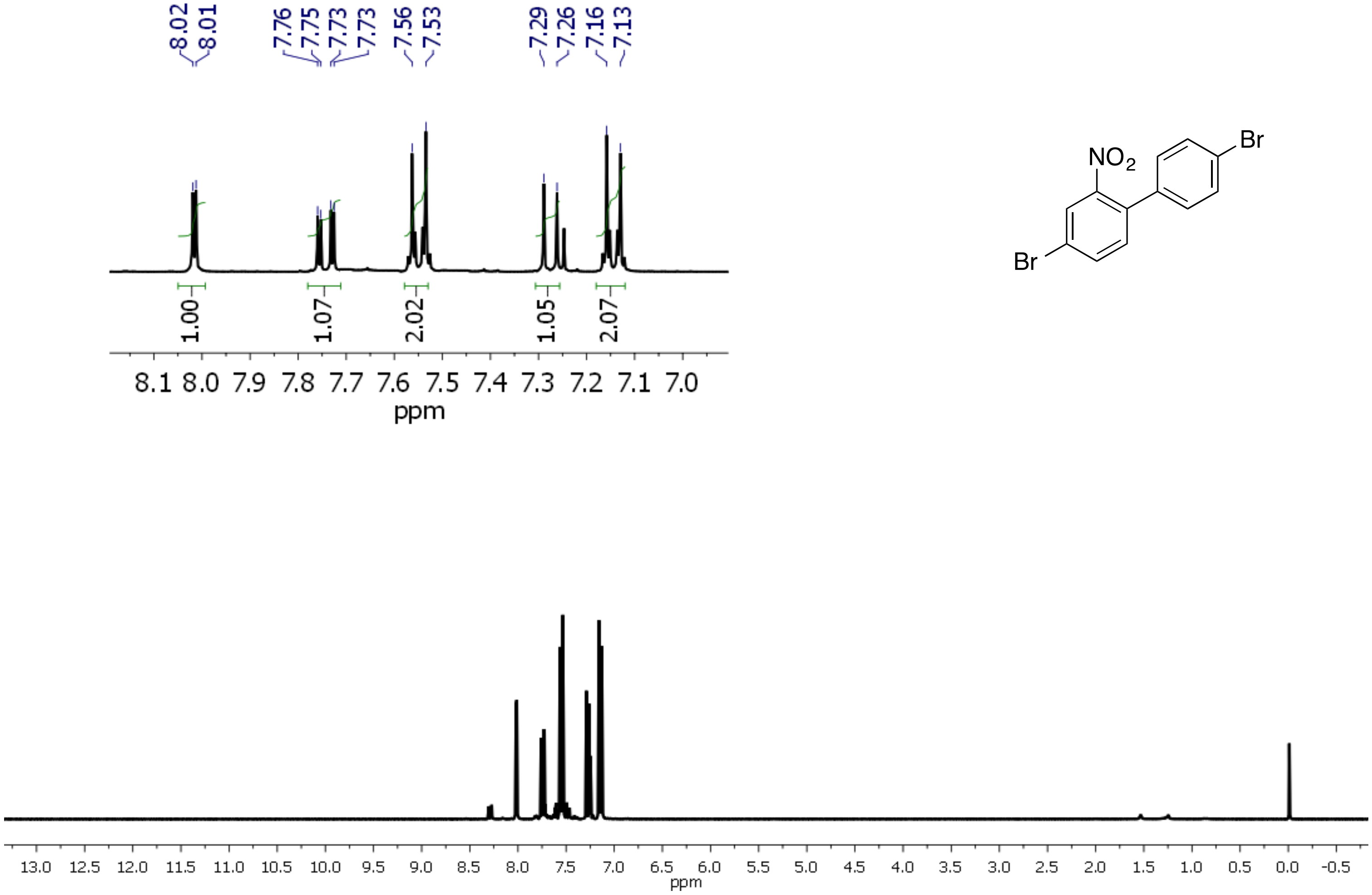

Figure S30. Spectrum of compound **10** (300 MHz, $CDCl_3$)

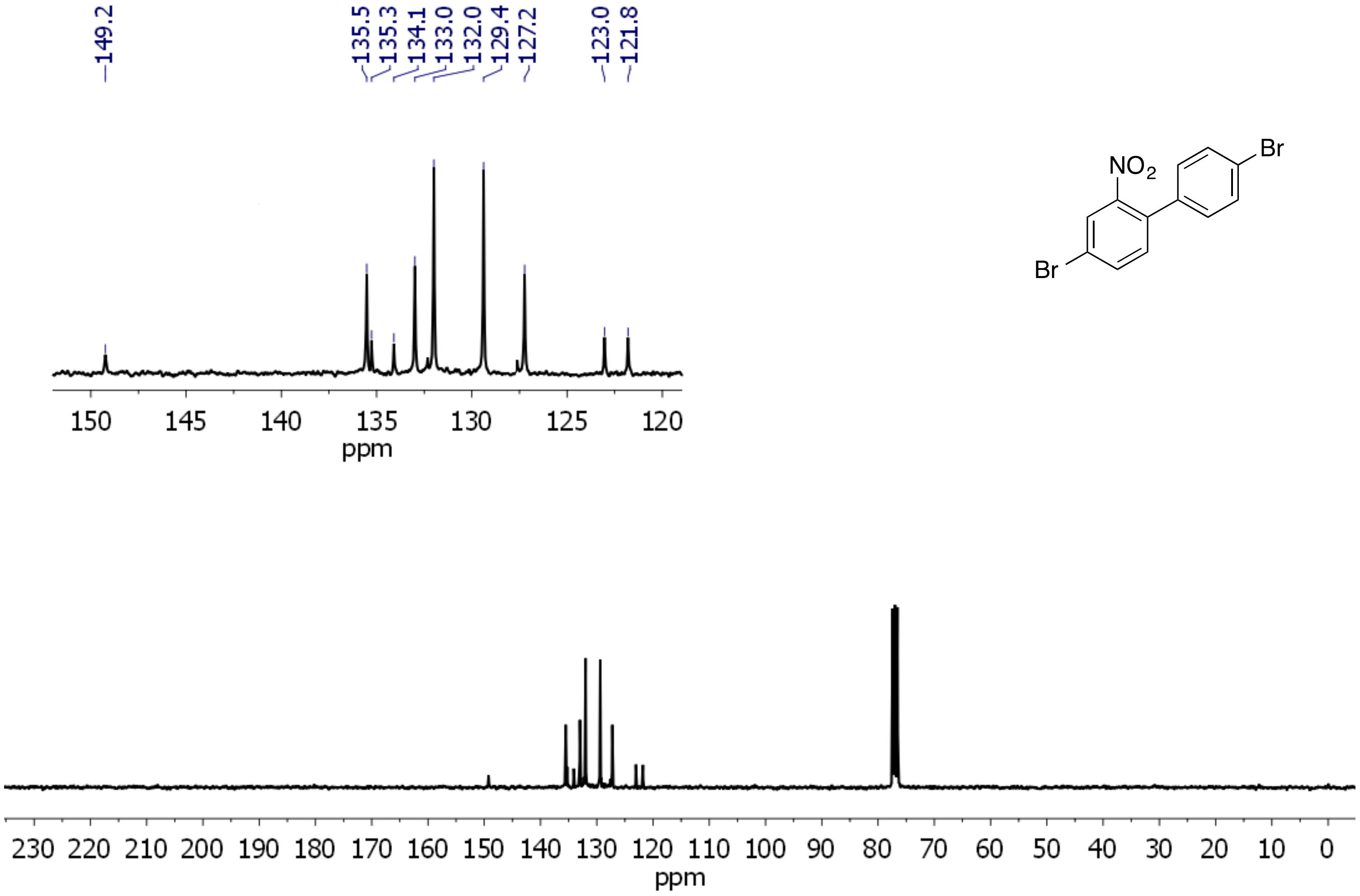

Figure S31. Spectrum of compound **10** (75 MHz, $CDCl_3$)

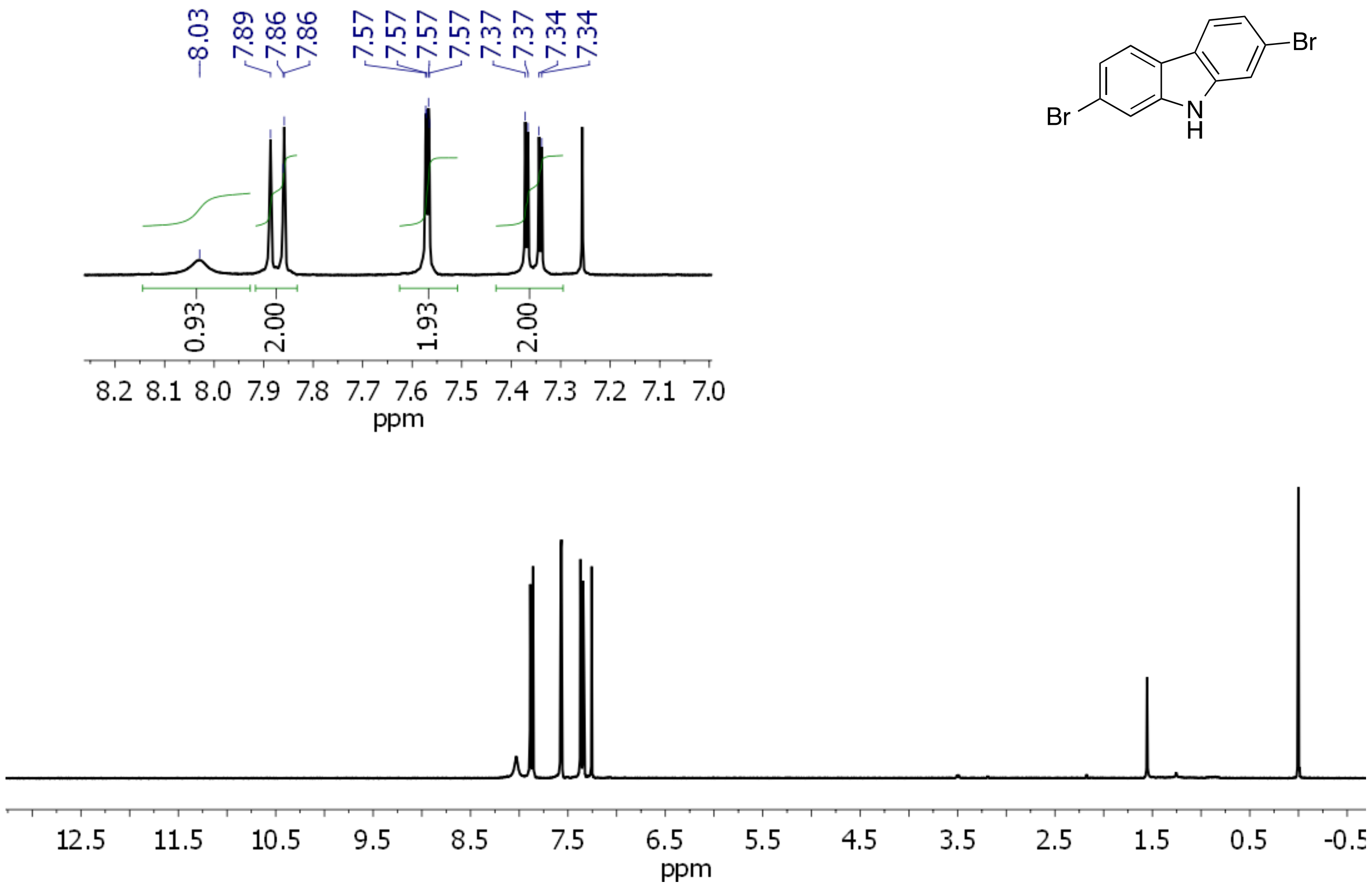

Figure S32. Spectrum of compound **9** (300 MHz, $CDCl_3$)

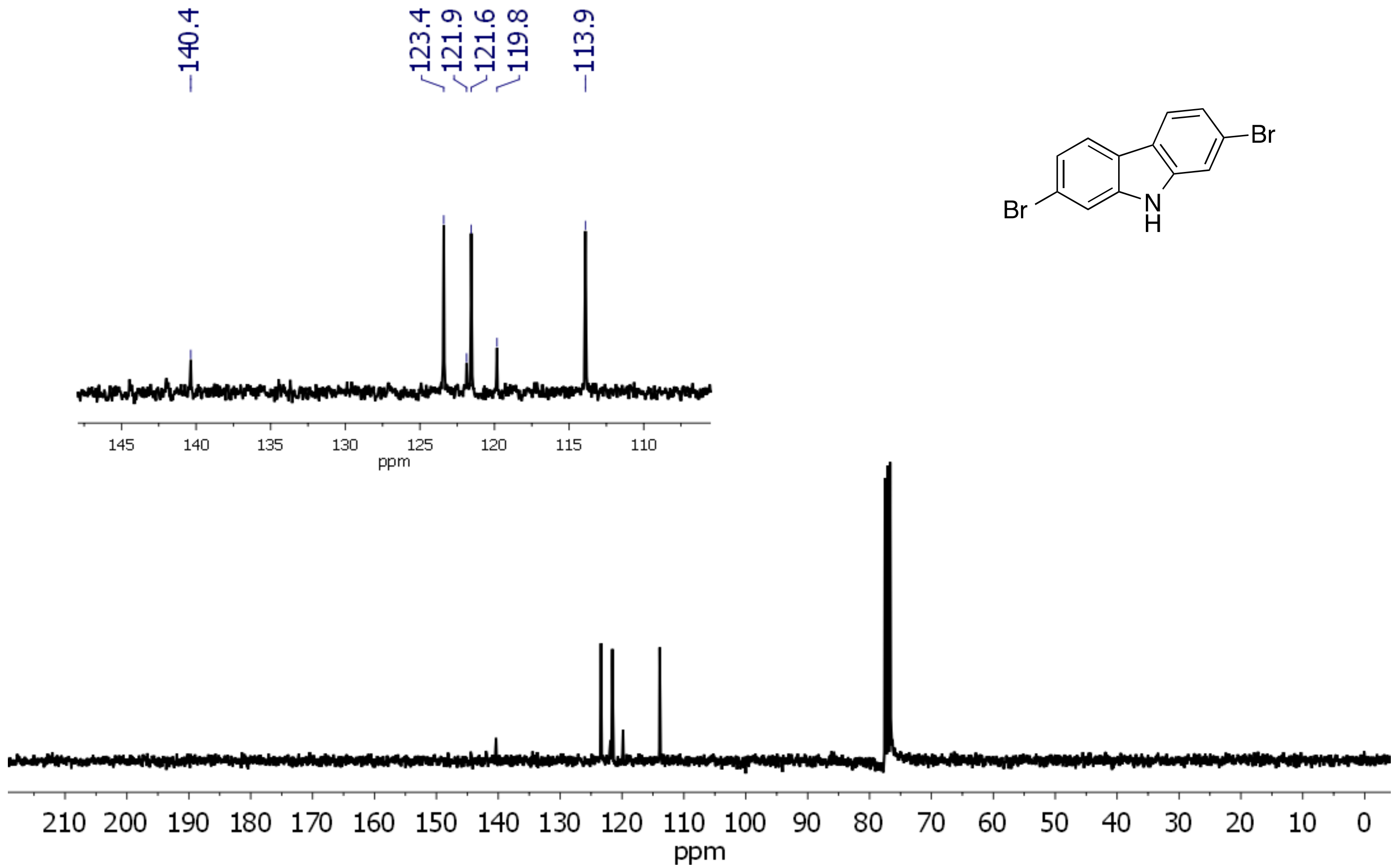

Figure S33. Spectrum of compound **9** (75 MHz, $CDCl_3$)

Thermal analyses

Differential scanning calorimetry and thermogravimetric analyses were carried out on a DSC analyzer Netzsch STA 449 F3 Jupiter under nitrogen atmosphere from 25 to 550 ºC with a heating rate of 10 ºC/min.

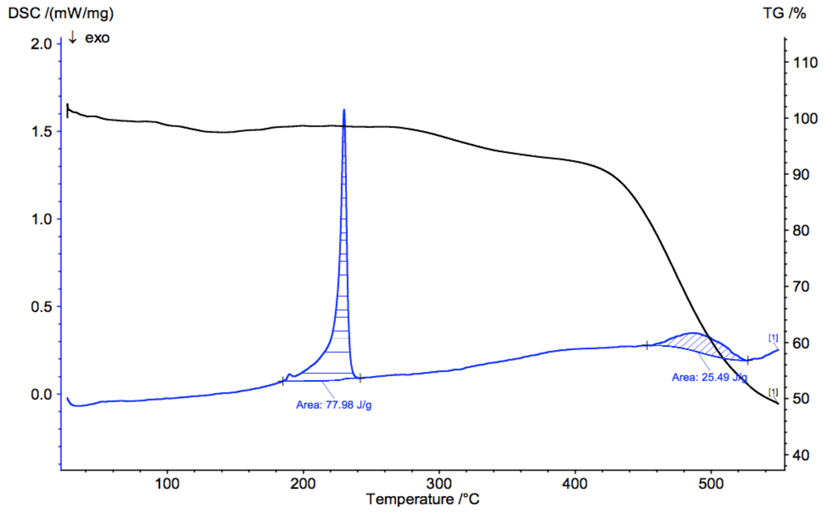

Figure S34.. Thermal analyses of compound **1:** (blue line) Differential scanning calorimetry (DSC); (black line) thermogravimetric analyses (TGA)

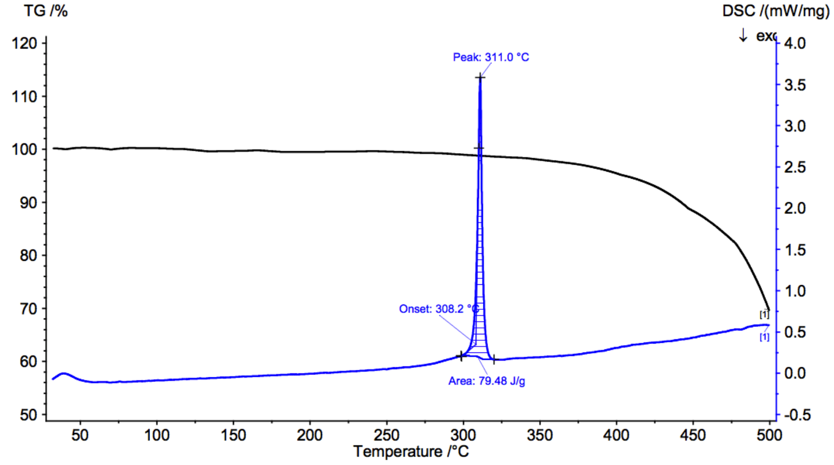

Figure S35. Thermal analyses of compound **2:** (blue line) Differential scanning calorimetry (DSC); (black line) thermogravimetric analyses (TGA)

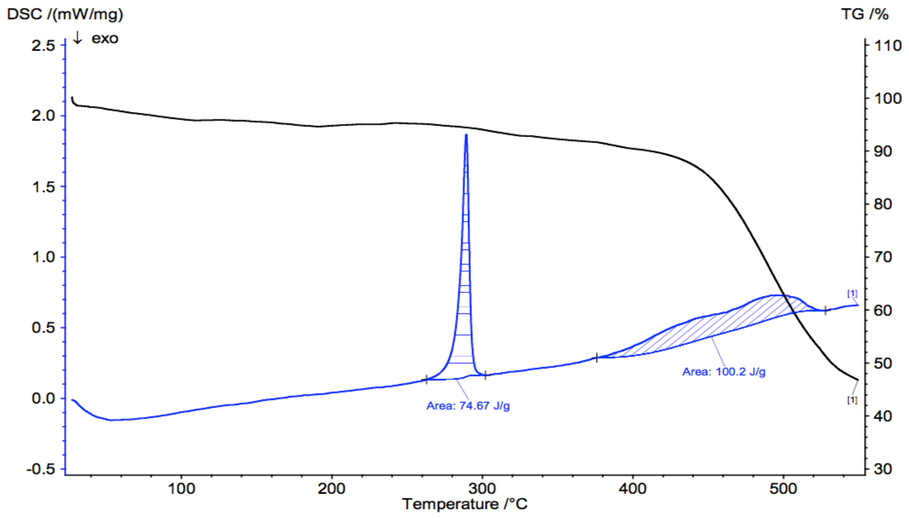

Figure S36. Thermal analyses of compound **3:** (blue line) Differential scanning calorimetry (DSC); (black line) thermogravimetric analyses (TGA)

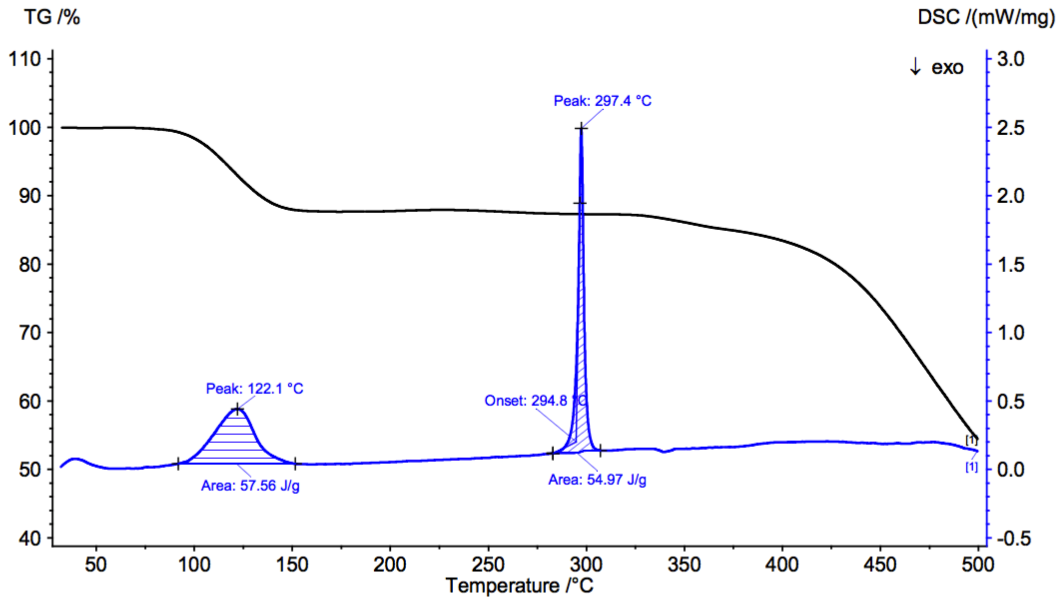

Figure S37. Thermal analyses of compound **4:** (blue line) Differential scanning calorimetry (DSC); (black line) thermogravimetric analyses (TGA)

Powder X-Ray diffraction

Analyses were carried out using Cu-$K_{\alpha 1}$ = 1.5406Å radiation, data were collected at room temperature in the range of 2Θ= 5-50° (step of 0.017°, step time 40.005 s), comparison can be seen from Figures S38 to S42.

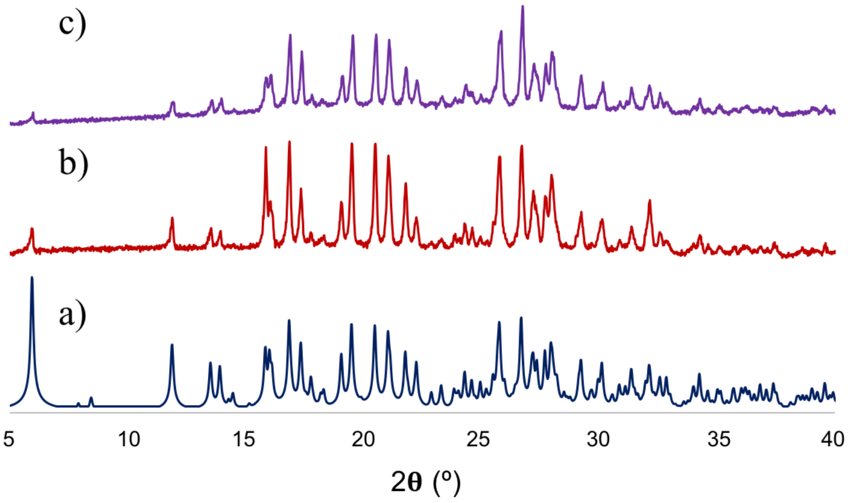

Figure S38. Comparison of powder X-ray diffraction patterns. a) Calculated from crystalline structure of **1** b) Experimental of **1** c) Experimental of **1-*d*₄**

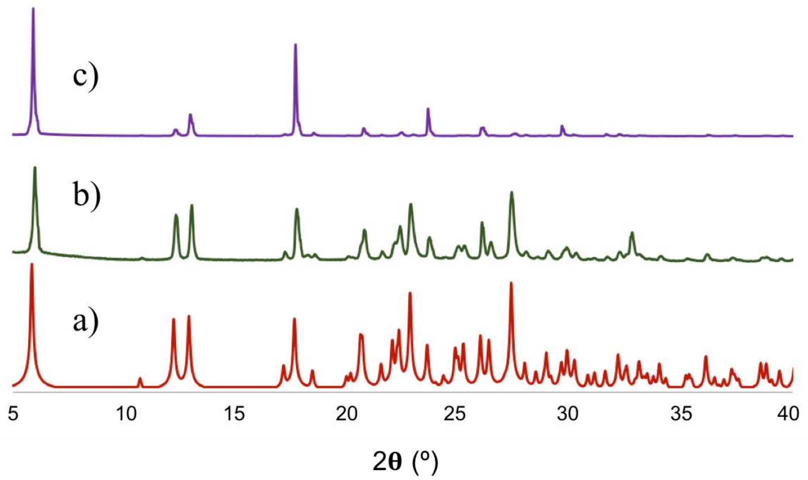

Figure S39. Comparison of powder X-ray diffraction patterns. a) Calculated from crystalline structure of **2** b) Experimental of **2** c) Experimental of **2-*d*₄**

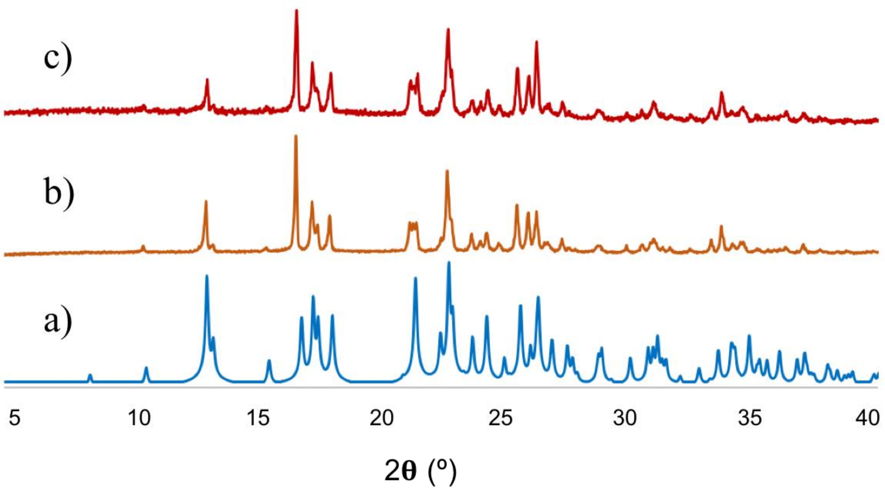

Figure S40. Comparison of powder X-ray diffraction patterns. a) Calculated from crystalline structure of **3** b) Experimental of **3** c) Experimental of **3-*d*$_4$**

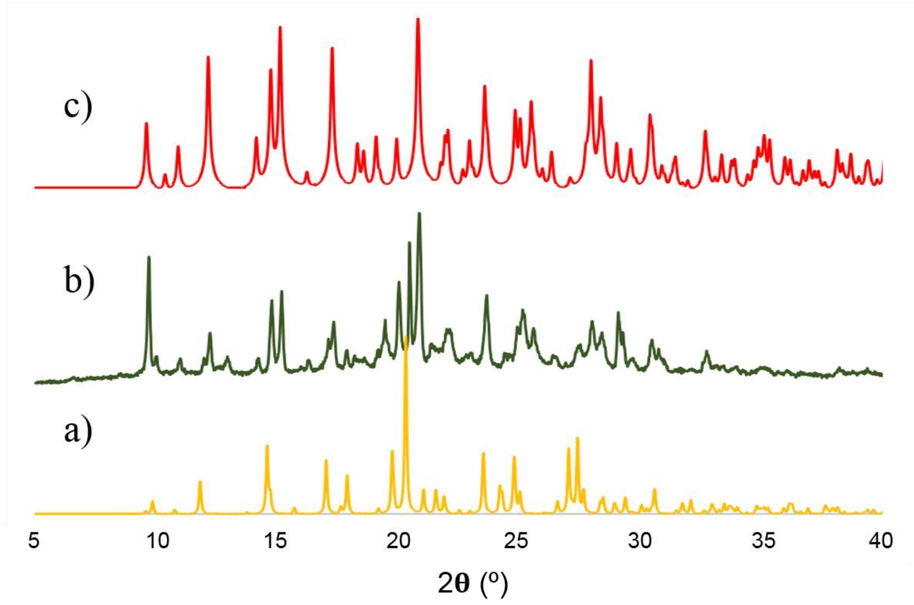

Figure S41. Comparison of powder X-ray diffraction patterns. a) Calculated from crystalline structure of **4** with DCM b) Experimental of **4** c) Calculated from crystalline structure of **4 solvent free**

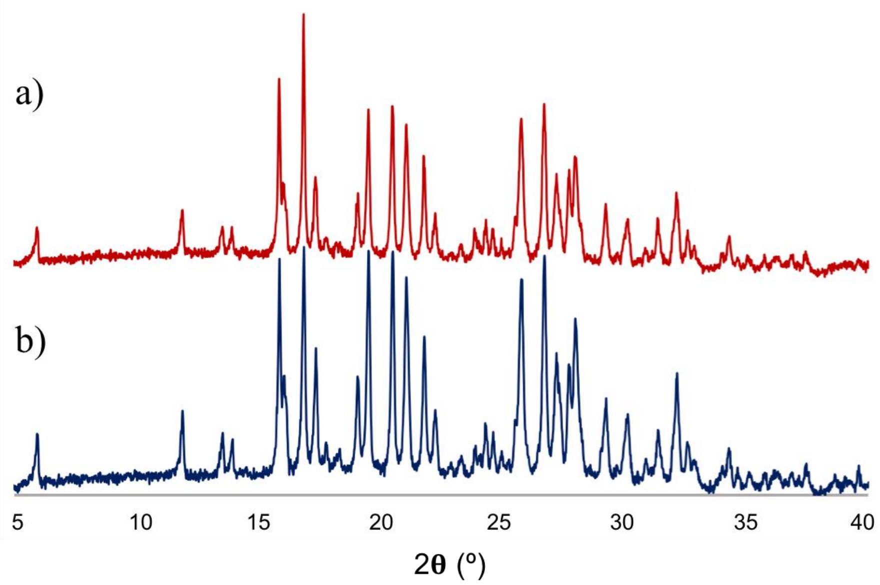

Figure S42. Powder X-ray diffraction patterns of compound **1** a) Freshly crystallized sample from DCM b) Sample after thermal treatment (370 K)

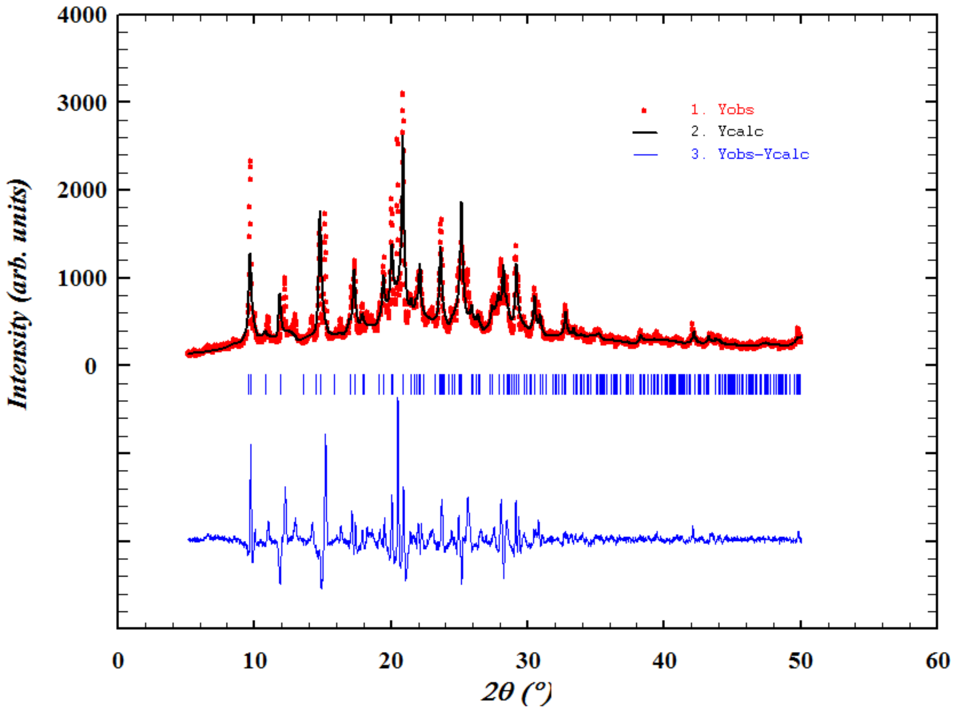

Figure S43. Le Bail adjustment of the experimental powder X-ray diffraction pattern for the bulk solid **4** using crystalline parameters of the solvate of **4**

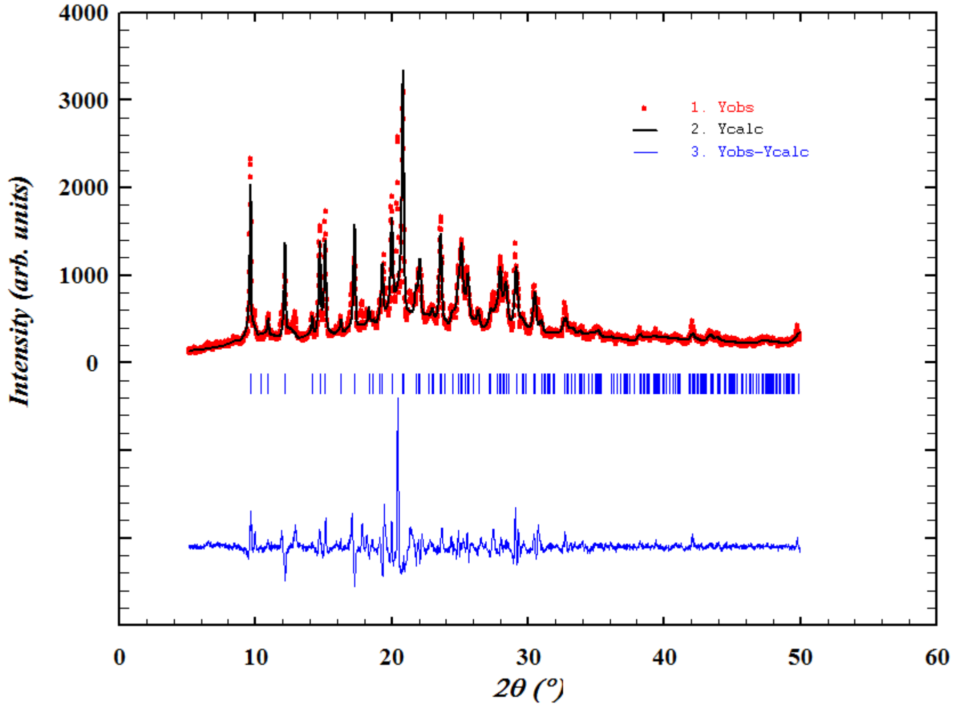

Figure S44. Le Bail adjustment of experimental powder X-ray diffraction pattern for the bulk solid **4** using crystalline parameters of **4 solvent free**

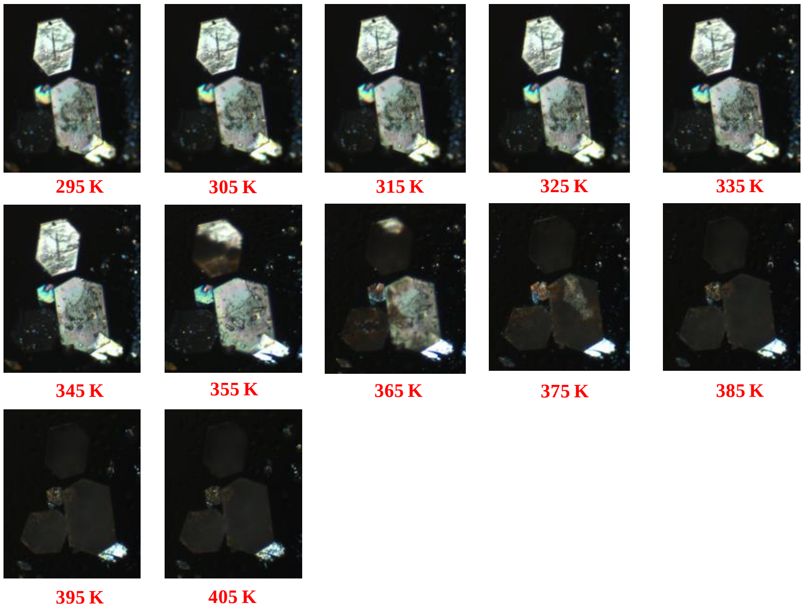

Figure S45. Phase transition in compound **4** from solvate form to thermal treated form analyzed under hot stage microscopy

Computational Details

Density functional theory (DFT) computations were carried out within the framework of the Projector-Augmented Wave (PAW) method,[5] as implemented in the Vienna Ab-initio Simulation Package (VASP).[6] The electron-ion interactions were described using the PAW potentials as are in the VASP library. The valence states were expanded in plane waves with an energy cut-off of 550 eV. All computations were done using the GGA-PBE exchange-correlation functional,[7] augmented by Grimme's D3-dispersion correction. [8,9] The Brillouin zone integration was performed using 2×1×1, 4×1×1, 2×2×2, and 2×1×2 Monkhorst-Pack k-point meshes. The structural parameters for each crystal were computed by a variable-cell optimization until all forces were smaller than 0.001 eV/Å.

Table S2. Comparison between experimental and computed lattice parameters for compounds **1-4**

**Lattice parameter**		**1**	**2**	**3**	**4**	**4 (solvent free)**
a (Å)	Exp.	7.0412	4.5701	11.1353	9.2718	9.2687
	Comp.	6.8306	4.4813	11.0089	9.1661	9.1332
b (Å)	Exp.	22.373	30.1408	13.5027	17.063	17.9615
	Comp.	21.992	29.9392	13.1848	16.6057	17.1722
c(Å)	Exp.	29.798	16.5121	8.2770	8.0988	8.2589
	Comp.	29.483	16.2760	8.0443	8.0627	8.1676
α (°)	Exp.	90.000	90.000	90.000	90.000	90.000
	Comp.	90.000	90.000	90.000	90.000	90.000
β (°)	Exp.	90.000	92.4100	109.6890	96.262	94.185
	Comp.	90.000	93.359	108.159	96.147	95.746
γ (°)	Exp.	90.000	90.000	90.000	90.000	90.000
	Comp.	90.000	90.000	90.000	90.000	90.000

Rotational Barriers

Figure S46 contains the computed energy barriers for compounds **2** and **3**, whose values were discussed in the main text. Figure S47 shows the computed rotational barriers for compound **4** with the DCM molecules included. The trend is that the presence of the solvent slightly increases the values of the energy barriers with respect to the solvent-free compound. For the case of the disrotatory motion, the most probable dynamic process, the highest energy barrier is increased from 29.2 kcal/mol to 30.5 kcal/mol due to the inclusion of the DCM molecules.

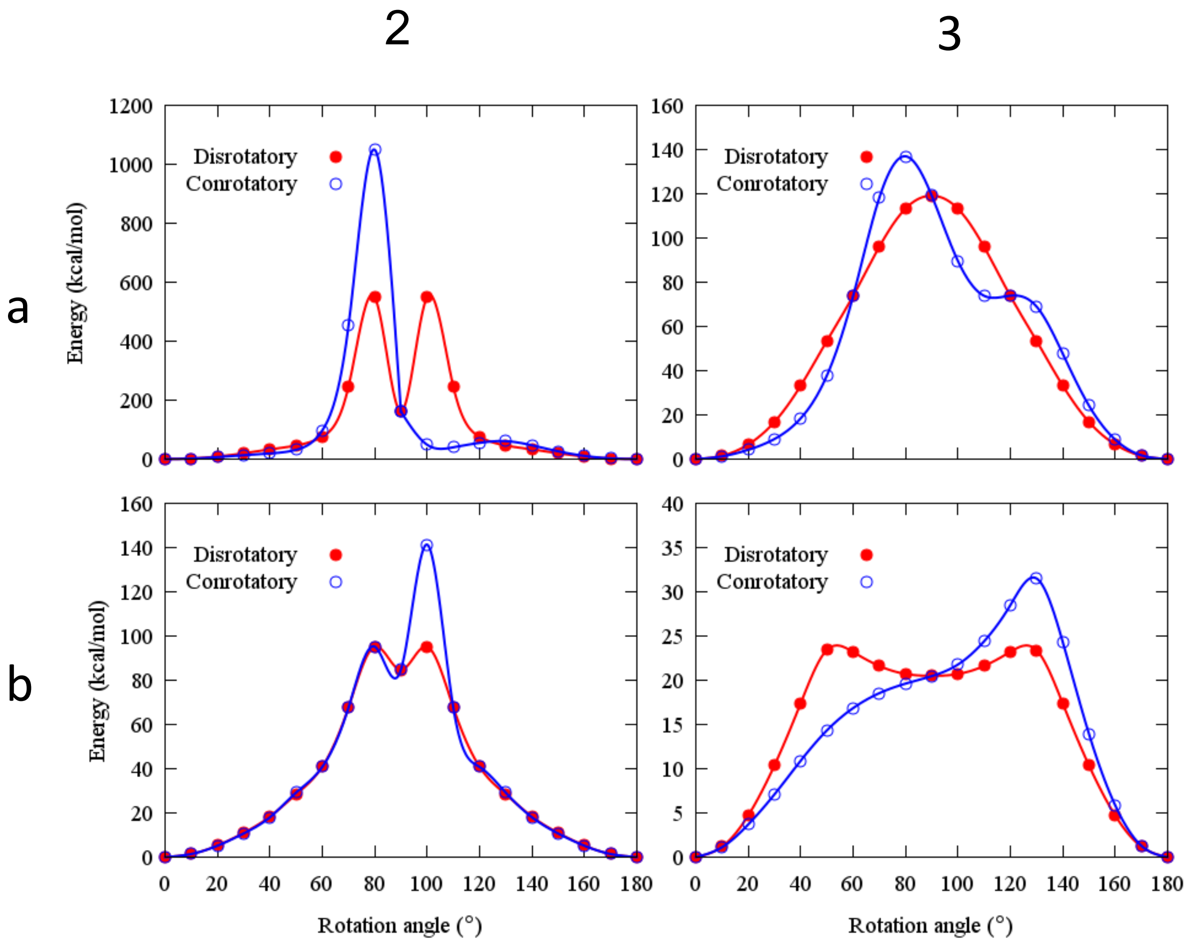

Figure S46. Rotational barriers for compound **2** and **3**. a) Rigid model; b) Relaxed model

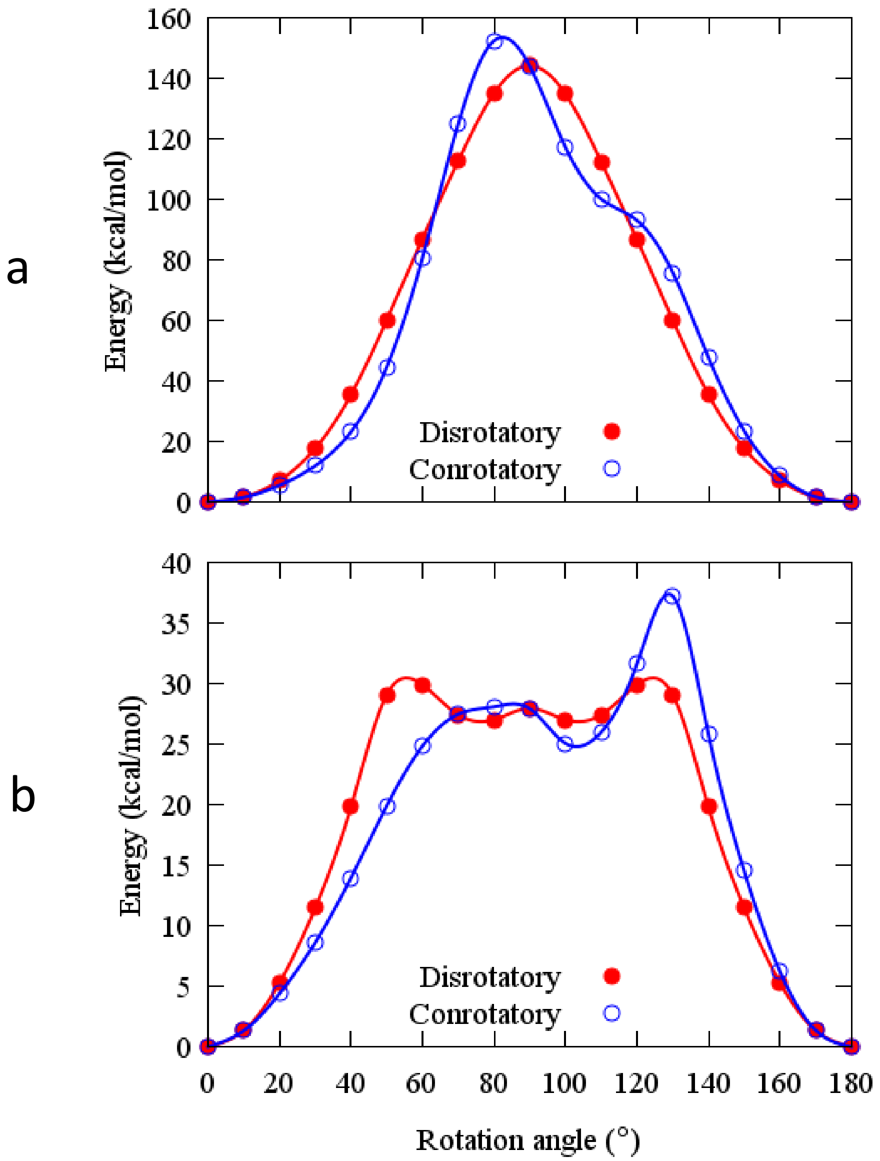

Figure S47. Rotational barriers for compound **4** with solvent included in the calculations. a) Rigid model; b) Relaxed model

References

S1. A. Enríquez-Cabrera, P. G. Lacroix, I. Sasaki, S. Mallet-Ladeira, N. Farfán, R. M. Barba-Barba, G. Famos-Ortiz, I. Malfant, *Eur. J. Inorg. Chem.* 2018, 531-543.

S2. H. Yu, J. Wang, X. Guo, R. Zhang, C. He, C. Duan, *Dalton Trans.*, 2018, **47**, 4040-4044.

S3. A. Aguilar-Granda, S. Pérez-Estrada, A. E. Roa, J. Rodríguez-Hernández, S. Hernández-Ortega, M. Rodríguez, B. Rodríguez-Molina, *Cryst. Growth Des.* 2016, **16**, 3435-344.

S4. A. Colin-Molina, S. Pérez-Estrada, A. E. Roa, A. Villagrana-Garcia, S. Hernández-Ortega, M. Rodríguez, S. E. Brown, B. Rodríguez-Molina, *Chem. Commun.*, 2016, **52**, 12833-12836.

S5. P.E. Blöchl, *Phys. Rev. B,* 1994, **50**, 17953-17979.

S6. G. Kresse, J. Furthmuller, *Phys. Rev. B,* 1996, **54**, 11169-11186.

S7. J. P. Perdew, K. Burke, and M. Ernzerhof, *Phys. Rev. Lett.*, 1996, **77**, 3865-3868.

S8. S. Grimme, J. Antony, S. Ehrlich, and S. Krieg, *J. Chem. Phys*. 2010, **132**, 154104.

S9. S. Grimme, S. Ehrlich, and L. Goerigk, *J. Comp. Chem.,* 2011, **32**, 1456.
